# Graphemic-phonetic diachronic linguistic invariance of the frequency and of the Index of Coincidence as cryptanalytic tools

**DOI:** 10.1371/journal.pone.0213710

**Published:** 2019-03-19

**Authors:** Vicente Jara Vera, Carmen Sánchez Ávila

**Affiliations:** Department of Applied Mathematics to Information Technology and Communications (Telecommunication Engineering), Polytechnical University, Madrid, Spain; Beijing University of Posts and Telecommunications, CHINA

## Abstract

Languages have inherent characteristics that make them their own and differentiated entities within their phyla and families. Even messages written in any language and later encrypted by cryptographic systems do not lose all of their characteristics, there remain aspects that help the cryptanalyst to recover them without knowing the decryption keys. For the characterization of the languages we will consider the frequencies of their graphemic and phonetic units and the Index of Coincidence, tools of fundamental utility in the field of Cryptography. Their diachronic invariance or survival over time in one language and their ability to discriminate against other languages will be analized. In order to do so, we will examine a total of 101 languages of which 261 texts have been taken. All of them are very diverse in style and time, taking us through a wide linguistic and temporal spectrum that will cover the period from the 6th century BC to the present day.

## Introduction

Cryptography is the applied science that designs and implements information protection systems by transforming the original messages in readable language into encrypted messages, impossible to decypher without the decryption key, although susceptible to cryptoanalytic attacks.

Natural languages are different from each other, in their lexicon and their syntax, their morphology or their phonology, among other aspects. Within Cryptography, encrypted texts which the cryptanalyst must deal with have gone through the disturbances and changes in the encryption that hide the information of their original message. However, as far as Cryptography is concerned, languages also show differences in their individual units, their graphemes or unigrams, as well as in their bigramas, trigrams, etc., and thus can be distinguished from each other [1]. It is, in short, something that Jaakub ibn Ishak al-Kindi (800–870) had already discovered and was independently rediscovered by Leon Battista Alberti (1404–1472) in 1466, as published in ´De Cifris´ (´On Encryption´) [2] [3] [4] [5] [6].

Cryptography divides its methods into transposition and substitution systems. Transposition takes the original message and changes the order of its units or minimum elements, be they characters or other symbols in which the message has been encoded. Thus, in the case of changing the characters for numbers in any numerical base, for example in base 2 or binary, in bits, either by ASCII, Extended ASCII, BCD, Unicode, etc., it would be a question of transposing bits (0 and 1) by changing them. It is, therefore, an algebraic permutation. On the other hand, the substitution takes the minimum number of units from the source text, or sets of minimum units, and substitutes them for other symbols or elements.

Even today's advanced cryptographic systems (symmetric cryptography of DES or AES, and asymmetric of RSA or Elliptic Curves, among others) are still based on transposition and substitution, whether using SBoxes, permutation functions, shifts, rotations or modular operations in an algebraic Group. And that is why transposition and substitution are linked to the concepts of confusion and diffusion, which Claude E. Shannon (1916–2001) defined as the basis of Cryptography [7].

Although classic systems, based on the graphemic units of languages, have not been used since the second half of the 20th century, at least as they had been used, because of the introduction of binary digital systems and the greater numerical processing capacity of recent computers, we cannot underestimate them, because some of them still remain unresolved. These include those using multiple substitution or homophony [8]; or some nomenclators, mixtures of substitutions, the insertion of nulls, homophony and dictionaries of substitution of common names, such as Manchester in 1783 [9], or Van Gelder in 1809 [10]; or one of the four cryptosystems housed in the ´Kryptos´ sculpture, created by Herbert James Sanborn, Jr. (1945-) in 1990 and placed at the entrance of the headquarters of the Central Intelligence Agency (CIA) in Langley (Virginia), based on classic ciphers (one of transposition and two of polyalphabetic substitution, all three solved), and pending the deciphering of the four cryptograms [11]. As we say, classic methods are the basis of all current systems [12].

With that said, and given the fundamental importance in the first place when solving the cryptosystem, to specify the original language of the clear or readable text that had been encrypted, it is necessary to use some basic tools to infer the language even on the basis of the text manipulated by the cipher system. The two fundamentals ones are the Frequency of their minimum units and the Index of Coincidence.

We begin by studying the frequency of the basic linguistic elements, which on the one hand will be the graphemic units or characters of a language, the letters, properly taken from languages written in romanized letters or as transcription; and on the other hand the phonetic units, a new aspect in our study which has not previously been considered, although useful and complementary, as we will see, and how necessary it would have been to decipher the texts sent during World War II by the United States Army with the help of Navajo Indians who transmitted military texts in their own language [4].

The study of frequency is fundamental for the cryptographic methods of transposition (groups, series, column/row, grid, etc.), since the alteration in the order of the elements does not modify the values of the units in their appearance in the ciphertext. This is the case, for example, even in such complex methods as the grid of Girolamo Cardano (1501–1576). On the other hand, in substitution systems the frequency is not altered in its numerical values, only in its assignments, which pass on to other symbols, at least in the monoalphabetic substitution systems where a source element is always replaced in the whole encryption process by the same target element. In the case of polyalphatic systems the frequency is less and tends to homogenize, dispersing the values.

The method of comparison or distance [13] between the frequency values of each gram (grapheme or phoneme), or multigram, will be of the Euclidean distance type (L_2_-norm), although, when estimating each unit in one dimension it is actually a Manhattan distance (L_1_-norm with a Minkowski index of p = 1):

$$\sum_{i=1}^{n}\left|BaseLanguage-{language}_{i}\right|$$

This formula must be understood in the sense that each language in our set will be compared to the remaining the languages, both in the different frequencies of graphemes and phonemes as a subtraction in absolute value.

On the other hand, and similarly to frequency, we will study the value of the Index of Coincidence (I.C.), Kappa value (Κ) [2] [14], which will help us to determine the language to which a certain text belongs, even if it had been encrypted by transposition or monoalphabetic substitution, the most common classic encryption types [2] [4] [6] [15]. Furthermore, the value of the I.C. will allow us to verify even if we are dealing with a polyalphabetic substitution cipher, allowing its value, if it is low, to suppose a polyalphabetic cipher and even to calculate the length of the key used in the system. Let us establish that in the case of a random text and with an alphabet of 26 characters the value of the I.C. is a scarce 0.0385, very remote data from what would be about double when some graphemic units are more common than others, as is usual in any language.

The value of the Index of Coincidence will be calculated in a general way without considering the number of units of the language in question as a normalizing aspect, making the calculations in accordance with the following expression, where ´n´ is the total amount of grams in all our set or sequence and ´f´ the number of occurrences of each gram, which varies from the unit to the maximum value ´m´ of different grams:

$$\sum_{i=1}^{m}\frac{f_{i}(f_{i}-1)}{n(n-1)}$$

The difference in the aforementioned frequency distance of the units is that in the case of the Index of Coincidence the frequency of occurrence of the pairings of these units is calculated, hence the order of the units in the sequence is indifferent. Thus, it is normal that the value of the I.C. is not as discriminatory in the languages and texts, as we will be able to verify, although it will also analyze its diachronic invariance.

On the other hand, for the two languages most widely considered here, Latin and Castilian Spanish, we will analyze their graphemic units (characters) and phonetic units (phonemes), to see their behavior over time. Subsequently we will compare them with other studied languages from which we will give their conclusive results. And in less detail we will do the same with about another hundred languages. In that way we will study whether there is a diachronic invariance in the grapheme and phonetic frequency of languages.

## Selected languages

From the total of phyla and languages [16] we will consider the Indo-European, Uralic, Altaic and Caucasian phyla as present on the great continent of Eurasia. In addition, we will study the Afro-Asiatic, Nilo-Saharan, Niger-Congo and Khoisan phyla from the African continent and Arabian peninsula. Next to them we must also consider the Austronesian Phylum, which extends from Oceania, Southeast Asia, Polynesia and the Pacific Islands to the island of Madagascar, a Phylum of which we will only take Malagasy language as a sample.

We will leave the following aside in this study: the Dravidian languages, from India, Sri Lanka, Nepal and Pakistan; the Eskimo-Aleut, Chukotko-Kamchatkan and Yeniseian languages, from Canada and the U.S.A. as well as the Asian Steppe, Russia and China; the Austroasiatic languages, from the East and Southeast of Asia, China and India; the Hmong-Mien Phylum and the Sino-Tibetan Phylum, from China and Tibet; the Na-Dene Phylum and the Algonquian, Chimakuan, Wakashan, Salishan, Siouan, Caddoan, Keresan, Iroquoian languages, as well as the Hokan Phylum and Muskogean, Penutian, Tanoan, Misumalpan, Yuki, Mayan, Oto-Manguean, Uto-Aztecan, Mixe-Zoquean, Huave, Totonacan, Chipaya-Uru, Gulf, Ge-Pano-Carib, Ecuadorian, Tunican, Chibchan-Paezan, Yok, Yanomaman, Mura or Andean languages, among others, present in all the extension from the north to the south of the American continent; the Papuan and Andamanese languages, from Papua New Guinea and Indonesia; and finally, the Australian languages, from the continent and islands of Oceania.

On the other hand, among languages not yet classified, we will take Basque and Etruscan into account [16].

In Table 1 we detail the number of languages and texts selected and analyzed for each Phylum.

**Table 1:** Phyla, languages and texts considered and studied.

PHYLUM	NUMBER OF LANGUAGES	NUMBER OF TEXTS
**Indo-European**	**42**	**126**
**Uralic**	**2**	**6**
**Altaic**	**2**	**6**
**Caucasian**	**3**	**6**
**Afro-Asiatic**	**26**	**57**
**Nilo-Saharan**	**12**	**21**
**Niger-Congo**	**10**	**27**
**Khoisan**	**1**	**3**
**Austronesian**	**1**	**3**
**Not classified**	**2**	**6**
**TOTAL**	**101**	**261**

**Fig 1:**
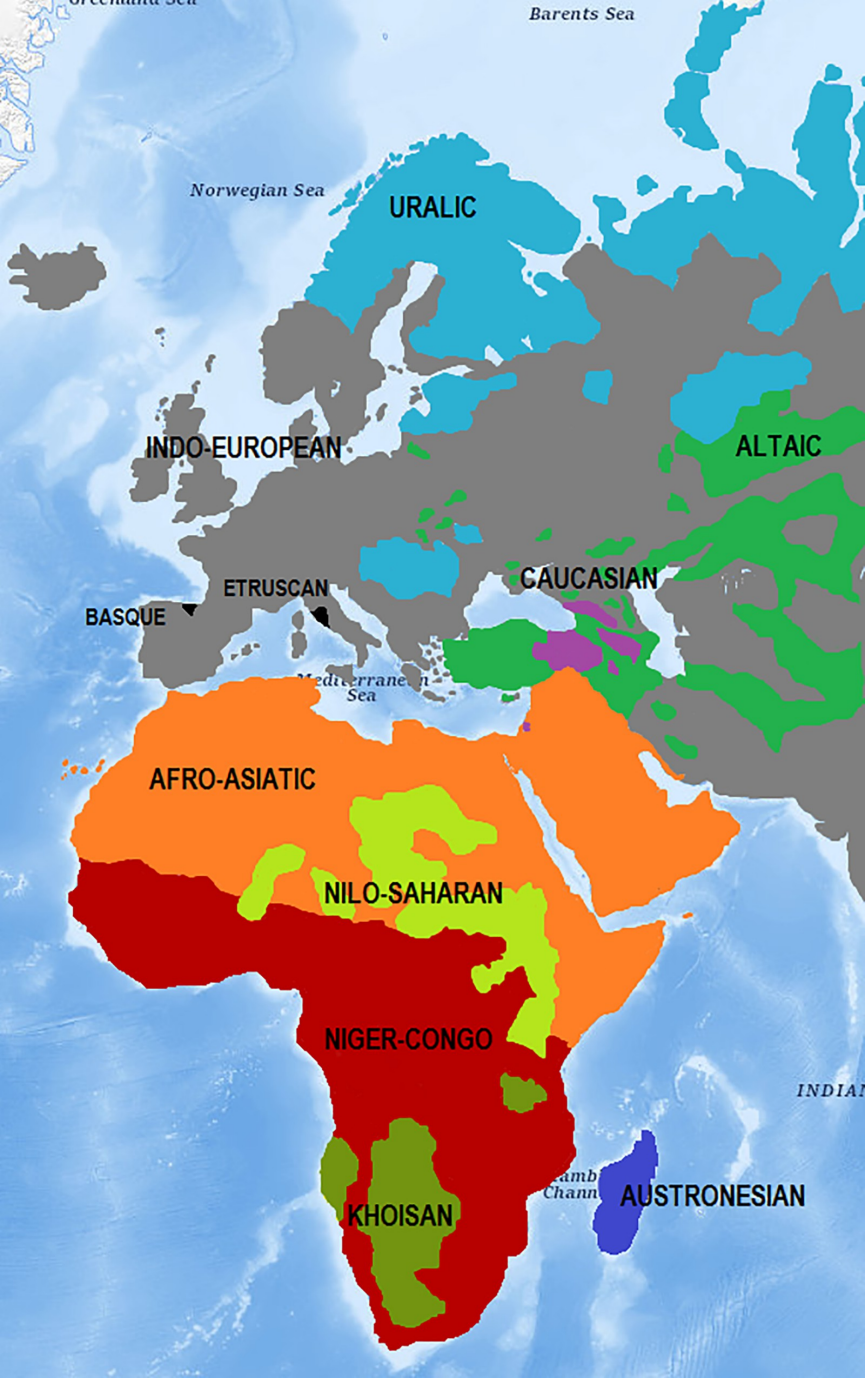
Map of linguistic phyla considered in their geographical environment: Europe, Africa and Western Asia [17] (modified figure, similar but not identical to the original for illustrative purposes).

The selected languages [12] are as follows (in which we will put the number of texts selected for each one in brackets). Fig 1 shows their geographical extension.

Indo-European Phylum: Latin (10), Tuscan (2), Lombard (1), Venetian (2), Napolitan (1), Sardinian (3), Romansh (2), Ladino (1), Galician-Portuguese (3), Catalan (1), Valencian (1), Balearic (1), Castilian (10), French (3), French-Provençal (3), Romanian (3), Prussian (3), Lithuanian (3), Polish (3), Croatian (3), Ukrainian (3), Welsh (3), Breton (3), Irish-Gaelic (3), Scottish-Gaelic (3), Icelandic (3), Danish (3), Norwegian (3), Swedish (3), Elfdalian (3), German (3), Dutch (3), Low German (3), English (3), Scottish (3), Frisian (3), Greek (3), Tsakonian (3), Albanian (3), Armenian (3), Romani (4) and Persian (3).

Uralic Phylum: Finnish (3) and Hungarian (3).

Altaic Phylum: Mongolian (3) and Turkish (3).

Caucasian Phylum: Abkhaz (2), Chechen (1) and Georgian (3).

Afro-Asiatic Phylum: Guanche (2), Awdjila (3), Sokna (1), Siwa (1), Kabyle (3), Tashelhiyt (2), Djerba (3), Djebel Nefusa (2), Ghomara (1), Ghadames (2), Tagargrent (3), Figuig (1), Tarifiyt (3), Chaouia (1), Tuareg Adagh (1), Tuareg Kidal (2), Tuareg Ayer (1), Zenaga (2), Punic (2), Hebrew (3), Arabic (3), Maltese (3), Hausa (3), Oromo (3), Coptic (3) and Wolaytta (3).

Nilo-Saharan Phylum: Jur Modo (1), Ma'di (2), Luo (3), Fur (2), Amdang (1), Kanuri (3), Masalit (1), Kibet (1), Maban (1), Zarma (3), Berta (1) and Gumuz (2).

Niger-Congo Phylum: Wolof (3), Shona (3), Zulu (3), Swahili (3), Yoruba (3), Ewe (3), Ibani (2), Kalabari (1), Moro (3) and Bambara (3).

Khoisan Phylum: Nama (3).

Austronesian Phylum: Malagasy (3).

Unclassified: Basque (3) and Etruscan (3).

The selection of texts, a total of 261 [12], covers a wide spectrum from the 6th century BC to the 21st century with the following temporal distribution: 1 text from the 6th century BC; 2 texts from the 3rd-2nd centuries BC; 2 texts from the 2nd century BC-the 1st century AD; 1 text from the 1st-2nd centuries; 1 text from the 2nd century; 1 text from the 2nd-4th centuries; 1 text from the 3rd-4th centuries; 1 text from the 4th century; 1 text from the 4th-5th centuries; 1 text from the 6th century; 1 text from the 7th century; 1 text from the 9th century; 1 text from the 11th century; 1 text from the 11th-12th centuries; 5 texts from the 12th century; 1 text from the 12th-13th centuries; 1 text from the 12th-14th centuries; 19 texts from the 13th century; 5 texts from the 13th-14th centuries; 2 texts from the 13th-15th centuries; 1 text from the 13th-17th centuries; 19 texts from the 14th century; 5 texts from the 14th-15th centuries; 26 texts from the 15th century; 1 text from the 15th-16th centuries; 1 text from the 15th-19th centuries; 28 texts from the 16th century; 10 texts from the 17th century; 2 texts from the 18th century; 1 text from the 18th-21st centuries; 22 texts from the 19th century; 57 texts from the 20th century; 4 texts from the 20th-21st centuries; 35 texts from the 21st century.

The 261 texts are composed of 177 in prose and 84 in verse to extend the linguistic spectrum of languages better.

## The graphemic and phonetic units of the selected languages

### Graphemes

The set of its graphemic elements has been considered for each language. All works were taken in their original spelling, regardless of the later versions that ´updated´ the texts. Languages use a romanized script in many cases. In the 14th century there were already a romanized orthography or extensions available on a Latin basis in many of the languages that we have considered, as well as in Celtic or Germanic languages, among others, in addition to graphemes of their own languages which are not of Latin origin. The same applies to Cyrillic languages. These differences were collected in the list of graphical symbols of each language in the particular study for each one [12].

In some cases, the linguistic material did not have a latinized or romanized base, although occasionally transliterated literary works have been used in a romanized alphabet, sometimes in critical editions by specialists in those languages. Although, in other cases we have made the transliterations ourselves.

Nevertheless, for the Indo-European, the Altaic, and the Caucasian, as well as the Greek Family of the Indo-European Phylum, and for the Semitic Family of the Afro-Asiatic Phylum, we have used their own orthographic signs of their alphabets as well as the romanized transliteration. We have done the same for languages such as Croatian and its Glagolitic and Cyrillic alphabet, or with Abkhaz, Chechen and Ukrainian, together with Cyrillic alphabets. In the case of Georgian we have again considered its own alphabet and its latinized transliteration. In the Armenian Family, for the language that gives it its name, we have counted again on its own alphabet and its transliteration to the romanized alphabet. Finally, for the Afro-Asiatic Phylum we have had to transliterate Coptic and Hebrew from their own alphabet, as well as Arabic, the latter language that will influence the Persian of the Indo-Iranian Subphylum, which we have analyzed both in its own alphabet and in its corresponding romanized transliteration.

In the case of Mongolian we did not have to consider the alphabets of its original works, since the material that was used in critical editions had already been transliterated.

For the set of phyla present on the African continent, the Austronesian Phylum, Niger-Congo, Nilo-Saharan, Khoisan and Afro-Asiatic (without considering Maltese, written in the romanized alphabet in the examples that have been considered; the aforementioned Arabic, Hebrew and Coptic), all their languages have been transliterated during the 19th and 20th centuries, predominantly, often following criteria close to those of the phonemes.

And finally, for Etruscan and Punic, two languages in use two millennia ago, although written with their own alphabets, we have been working with specialized critical works and their already-made romanized transliterations.

### Phonemes

We have made a correspondence of each graphemic unit, or units, if there is more than one, together with their unit or phonetic units by means of their transcription in all of the languages that we have selected. If we have talked before about differences between alphabets and their formation throughout history and their modifications, now we will move within the same domain into the sounds, the phonemes, the minimum units of the phonetic set for each language, which will not always be easy for many languages.

We have followed the characterization of the International Phonetic Association (IPA), which will serve as a common framework for all of the languages, at the same time as homogenizing and normalizing [18].

The phonetic analysis is a novelty in our cryptological study, compared to that of the usual grapheme, for the characterization of a language.

The examination of languages and texts has offered a total of 206 different graphemic units (Table 2). The number of different phonetic units is about 183 (Table 3).

**Table 2:** Total set of graphemes.

**a**	**b**	**c**	**d**	**e**	**f**	**g**	**h**	**i**	**j**	**k**	**l**	**m**	**n**	**ñ**	**o**	**p**	**q**	**r**	**s**
**t**	**u**	**v**	**w**	**x**	**y**	**z**	**à**	**á**	**â**	**ä**	**ã**	**å**	**ă**	**ā**	**ą**	**ą̊**	**a:**	**ʌ**	**ʌ:**
**æ**	**è**	**é**	**ê**	**ě**	**ë**	**ẽ**	**ė**	**ẹ**	**ĕ**	**ē**	**ę**	**e:**	**ə**	**ə:**	**ɛ**	**ɛ̃**	**ɛ:**	**ì**	**í**
**î**	**ï**	**ĩ**	**ị**	**ī**	**į**	**i:**	**ɨ**	**ı**	**ɪ:**	**ò**	**ó**	**ő**	**ô**	**ö**	**õ**	**ọ**	**o̩**	**ǫ**	**o:**
**ɔ**	**ɔ̃**	**ɔ:**	**œ**	**ù**	**ú**	**ű**	**û**	**ü**	**ũ**	**ụ**	**ū**	**ų**	**u:**	**ʊ**	**ʊ:**	**ḅ**	**ɓ**	**ß**	**ć**
**c̔**	**č**	**ċ**	**č̣**	**c̄**	**ḍ**	**ḏ**	**d̥**	**ɗ**	**ɖ**	**ð**	**ƒ**	**ǧ**	**ġ**	**ğ**	**ɣ**	**ḥ**	**h̬**	**ḫ**	**ẖ**
**ћ**	**ĵ**	**ǰ**	**ɟ**	**k̔**	**k̄**	**k̢**	**ƙ**	**ł**	**m̩**	**ń**	**n̩**	**ŋ**	**ŋ̩**	**ɲ**	**p̔**	**ṗ**	**q̇**	**ř**	**ṙ**
**ṛ**	**ɽ**	**ɿ**	**ś**	**š**	**ṣ**	**ş**	**ʃ**	**ʄ**	**ſ**	**t̔**	**ṭ**	**ț**	**ṯ**	**t̪**	**t̢**	**ŵ**	**x̢**	**ý**	**ŷ**
**ÿ**	**ȳ**	**ź**	**ž**	**ż**	**ẓ**	**ƶ**	**ʒ**	**ƹ**	**ç**	**ċ̨**	**ҫ̇**	**θ**	**ξ**	**σ**	**ʋ**	**φ**	**ø**	**χ**	**þ**
**Ь**	**Ъ**	**Ӵ**	**C**	**K**	**L**	**N**	**T**	**7**	**ʰ**	**ʲ**	**ʷ**	**ǀ**	**ǁ**	**ǂ**	**ǃ**	**ʕ**	**ʔ**	**ʢ**	**ʡ**
**'**	**ʻ**	**ʼ**	**ᶜ**	**ᵓ**	°														

**Table 3:** Total set of phonemes.

**a**	**a:**	**ã**	**ɐ**	**ɐ̯**	**ɑ**	**ɑ:**	**ɑ̃**	**æ**	**æ:**	**e**	**e:**	**ë**	**ẽ**	**ẽ:**	**ə**	**ə:**	**ɵ**	**ɛ**	**ɛ:**
**ɛ̃**	**ɝ**	**i**	**i:**	**ï**	**ĩ**	**ĭ**	**i̯**	**ɪ**	**ɪ:**	**ɨ**	**o**	**o:**	**ö**	**õ**	**õ:**	**ɔ**	**ɔ:**	**ɔ̃**	**ø**
**ø:**	**ɒ**	**ɒ:**	**œ**	**œ:**	**œ̃**	**u**	**u:**	**ũ**	**ŭ**	**u̯**	**ʉ**	**ʉ:**	**ʉ̃**	**ʊ**	**ʊ:**	**b**	**b:**	**b°**	**b̥**
**b̤**	**ɓ**	**β**	**c**	**c:**	**ç**	**ɕ**	**d**	**d:**	**d°**	**d̥**	**d̤**	**d̴**	**d̪**	**ɗ**	**ɖ**	**ð**	**f**	**g**	**g:**
**g°**	**ɠ**	**ɢ**	**ɣ**	**ɣ:**	**h**	**h̃**	**ɦ**	**ɧ**	**ħ**	**ɥ**	**j**	**j:**	**ʝ**	**ɟ**	**ʄ**	**k**	**k:**	**l**	**l:**
**l̥**	**ɫ**	**ɬ**	**ɭ**	**ʎ**	**m**	**m:**	**m̤**	**m̩**	**ɱ**	**n**	**n:**	**n̩**	**ŋ**	**ŋ̩**	**ɲ**	**ɴ**	**p**	**p:**	**ɸ**
**q**	**q:**	**r**	**r:**	**r̥**	**r̴**	**r̪**	**ɾ**	**ɽ**	**ɹ**	**ʀ**	**ʁ**	**s**	**s:**	**s̪**	**s̺**	**ş**	**s̴**	**ʂ**	**ʃ**
**t**	**t:**	**t̥**	**t̪**	**t̴**	**ʈ**	**θ**	**v**	**ʌ**	**ʌ:**	**ʋ**	**w**	**w:**	**ɯ**	**ɯ:**	**x**	**χ**	**y**	**y:**	**ʏ**
**ʏ̃**	**z**	**ʐ**	**z̴**	**ʑ**	**ʒ**	**ʒ°**	**ʕ**	**ʔ**	**ˤ**	**ʢ**	**ʡ**	**ǀ**	**ǁ**	**ǂ**	**ǃ**	**ˠ**	**ʰ**	**ʲ**	**ⁿ**
**ʷ**	**'**	**ŋ°**																	

The number of units for the 261 texts of the 101 languages has a Mean of μ = 3784.63 for the graphemes and of μ = 3790.09 for the phonemes.

## Latin and Spanish (a detailed analysis)

Among the 101 languages we will take the case of Latin and Spanish or Castilian to highlight their selection of texts in greater detail and study the variations in the graphemic and phonetic values of their Frequency and their Index of Coincidence over time.

If, on average, 2.58 texts/language have been taken, in the case of these two languages we have gathered 10 texts. The stability of the values of Frequency and I.C., after the results with Latin and Spanish, as will be verified, led to a smaller number of texts in the rest of the languages (usually 3 or 2 texts per language).

### Latin

Latin is the language that gives rise to the entire Romance Family, although it is situated in the Indo-European Phylum, Italic Subphylum, Latino-Faliscan Family. It is an unique case, as the origin of a whole linguistic family is perfectly known [16] [19]. Its geographical extension corresponded to the entire Roman Empire in the West during the Middle Ages, although limited to political and educated circles, and it was the language used in the courts and chancelleries of all Europe, in the palatine and catedral universities of the nascent states, as well as Catholic liturgical language and usual in religion.

We will take 10 texts in prose and verse in a wide range of the literary history of the Latin language for our analysis, from the 1st century until the 14th [20] [21] [22].

#### Selection of texts

**I-II centuries. Text in verse.** We will begin with a work in verse by Decimus Iunius Iuvenalis (ca. 60- ca. 130), in particular his ´Satura I´ (´Satire I´), which we can place between the end of the 1st century and the beginning of the 2nd century of our era [23] [24].

**IV century. Text in prose.** The following text will be the main pericopes of Saint Luke's Gospel in which the Virgin Mary is the main protagonist, which are the following fragments: the Presentation of the Lord in the Temple (Lk 2,21–39), the Annunciation (Lk 1,26–38), the Visitation (Lk 1,39–56) and the Birth in Bethlehem (Lk 2,1–20). The chosen text will be the version of the ´Vulgate´, made by Saint Ieronimus (ca. 340–420) at the end of the 4th century [25] [26] [27]. Although there have been many other versions over the following centuries, even with interpolations and copyist errors, we will make use of a critical version such as the so-called ´Nova Vulgata´ (´New Vulgate´) from the year 1974 by José Mª Valente Bover, S.I. (1877–1954) and Joseph O'Callaghan, S.I. (1922–2001) [28] [29].

**IV-V centuries. Text in prose.** We will take our next text from a classic author and widely read in the Middle Ages, as is Saint Aurelius Augustinus Hipponensis (354–430), who had also a profound knowledge of this language [30]. We will consider his work ´Confessionum´ (´Confessions´), written between the years 397 and 401, the I to X fragments of the book I [31] [32].

**VI century. Text in verse.** We will take a serie of poems by the poet Venantius Fortunatus (ca.530- ca.600) [22], such as the hymn dedicated to the Holy Cross, ´Hymnus in Honore Sanctae Crucis´ (´Hymn in Honor of the Holy Cross´), another one to the Church of Paris, ´De Ecclesia Parisiaca´ (´On the Church of Paris´), and the famous ´Pange, Lingua´ (´Sing, my Tongue´), to the Body of Christ, all of them from the second half of the 6th century [33].

**VII century. Text in prose.** From Saint Isidorus Hispalensis (ca.556-636) [34] we will select the book IV, which is about Medicine (´De Medicina´) of his encyclopaedia ´Etymologiarum Libri Viginti Sive Origines´ (´Twenty Books on Etymologies or Origins´), written during the last 20 years of his life [35].

**XI-XII centuries. Text in verse.** From the anonymous chants of ´Carmina Burana´ (´Songs from Beuern´), satirical and loving goliard ballads, we will take chant number 60, of amorous style, which could be dated from around the 11th and 12th centuries [36] [37].

**XII century. Text in verse.** We will take the hymn ´Ad Vesperas´ (´For Vespers´) and the ´Hymnus Gratiarum post Epulas´ (´Thanksgiving Hymn After the Banquet´) by Petrus Abelardus (1079–1142) [38], leading intellectual figure of the 12th century, written between the years 1130 and 1135 [22].

**XIII century. Text in prose.** From a very famous preacher of his time throughout Europe, the franciscan Saint Antonius Patavinus, O.F.M. (1195–1231) [39], we will take a sermon for the feast of the Candelaria, ´In Purificatione Beatae Mariae Virginis´ (´In the Purification of the Blessed Virgin Mary´), from the 13th century [40].

**XIII century. Text in prose.** Our next literary work corresponds to a fragment of the theological composition most studied and read during the Middle Ages, therefore, basis of discussions, arguments and a way of understanding and expressing the Latin language for centuries. This is the ´Summa Theologiae´ (´Compendium of Theology´) by Saint Thomas Aquinas, O.P. (1225–1274) [41]. We will select a fragment of part I, in particular question II in its proemium and articles I to III (I q. II a. I-III), which we know were written between 1266 and 1268 [42].

**XIV century. Text in verse.** We will choose our last text by the poet Francesco Petrarca (1304–1374) [43]. It will be one of his songs written in Latin of his ´Bucolicum Carmen´ (´Bucolic Song Book´), written between 1346 and 1357, specifically the first eclogue, ´Parthenias´ (´Virgin´) [44].

#### Graphemic-phonetic correspondences

Carrying out the linguistic analysis for the 10 selected texts, both in their graphemic and phonological aspects [45], our results regarding their correspondence are represented in Table 4:

**Table 4:** Latin language. Latin alphabet (lowercase letters) and phonetic transcription (IPA symbols).

LATIN ALPHABETIC CHARACTERS (LOWERCASE)																								
IPA PHONETIC TRANSCRIPTION																								
**A**	**b**	**c**	**d**	**e**	**f**	**g**	**h**	**i**	**J**	**k**	**l**	**m**	**n**	**o**	**p**	**q**	**r**	**s**	**t**	**u**	**v**	**x**	**y**	**z**
**A**	**b**	**k/tʃ**	**d**	**ɛ**	**f**	**g/ɲ**	**∄/f/ʰ**	**ɪ**	**ɪ**	**k**	**l**	**m**	**n/ɲ**	**ɔ**	**p/f**	**k**	**r**	**s**	**t/tʰ**	**ʊ/w**	**v/w**	**ks**	**i**	**z**

NOTE: The consonant <c> preceded by <e>, <i>, <ae> or <oe> adopts the /tʃ/ sound. In other cases it is pronounced /k/. The set <gn> takes the sound /ɲ/. In other cases <g> becomes /g/ and <n> becomes /n/. The set <ph> adopts the sound /f/. In other cases the sound <p> is /p/ and <h> is phonetically muted. The block <th> becomes /t^h^/. In other cases <t> becomes /t/. The vowel <u> preceded by <q> becomes /w/. In other cases <u> takes the sound /ʊ/. The consonant <v> preceding <g>, <q> or <s> takes the /w/ sound. In other cases it becomes /v/.

### Spanish (Castilian)

Spanish or Castilian is a language that belongs to the Indo-European Phylum, Italic Subphylum, Romance Family, Western Subfamily, Galo-Iberian-Romance Group, Ibero-Romance Subgroup, Spanish Area [16] [46].

Our selection of its vast literary history, in this case, as for Latin, will be a total of 10 texts, taking works in prose and verse from the 13th to the 20th century for our analysis [47] [48] [49] [50] [51] [52].

#### Selection of texts

**XIII century. Text in verse.** We will start by taking a text from the 13th century selected from the work by Gonzalo de Berceo (ca.1199-ca.1255) [47], in particular, the first three miracles of ´Milagros de Nuestra Señora´ (´Miracles of Our Lady´) (ca.1246-1252): ´La casulla de San Ildefonso´ (´Saint Ildefonso's Chasuble´), ´El sacristán fornicario´ (´The Fornicator Sacristan´) and ´El clérigo y la flor´ (´The Clergyman and the Flower´), the stanzas from 47 to 115 [53] [54].

**XIV century. Text in prose.** For the 14th century we will take the work ´Libro de los Enxiemplos del Conde Lucanor et de Patronio´ (´Book of the Examples of Count Lucanor and Patronio´) by the Infante Juan Manuel (1282–1348), written in prose around 1330 as an example [47] [55]. We will select the enxiemplo I in its folios from 6 to 8 [56].

**XIV century. Text in verse.** As a poetic text from the 14th century we will choose the ´Libro del Buen Amor´ (´The Book of Good Love´) by Juan Ruiz, Archpriest of Hita (died ca.1351), dated around 1330 or 1343 [47] [57]. We will consider the verses that include from ´Del Ave María de Santa María´ (´Ave Maria of Santa Maria´) and the following four blocks of ´Cantica de loores de Santa María´ (´Song of Praise of Santa Maria´), that is, the verses from 1661 to 1689 [58].

**XV century. Text in verse.** We will choose the poetic work ´Las coplas por la muerte de su padre´ (´The Couplets on the Death of his Father´) (ca.1476), folios 8 to 42 [59], by Jorge Manrique Toletanus (1440–1479) [47] [60].

**XV century. Text in prose.** From Fernando de Rojas (ca.1476-1541) [47] [61] we will take his work ´Libro de Calixto y Melibea y de la Puta Vieja Celestina´ (´Book of Calixto and Melibea and the Old Whore Celestina´) (1499), in particular the folios from 8 to 24, Act I, an example in prose from the 15th century [62].

**XVII century. Text in prose.** Already in the 16th century we will select ´El ingenioso hidalgo don Quixote de la Mancha´ (´The Ingenious Hidalgo Don Quixote of La Mancha´), by Miguel de Cervantes Saavedra (1547–1616) [49] [63] in 1605, chapter I, which includes the folios from 1v to 4v [64].

**XVIII century. Text in verse.** We will go on to the 18th century with Tomás de Iriarte y Nieves Ravelo (1750–1791) [65], from whom we will take his poetic work, more specifically, from the genre of the fable, his ´Fábulas literarias´ (´Literary Fables´), from 1782. We will specifically select the first four: ´El elefante y otros animales´ (´The Elephant and Other Animals´), ´El gusano de seda y la araña´ (´The Silkworm and the Spider´), ´El oso, la mona y el cerdo´ (´The Bear, the Monkey and the Pig´) and ´La abeja y los zánganos´ (´The Bee and the Drones´) [66].

**XX century. Text in verse.** In the poetry of the 20th century we have Vicente Aleixandre y Merlo (1898–1984) [67] [68] and we will extract the following poems from his poetic work ´Espadas como labios´ (´Swords Like Lips´), from 1932: ´Mi voz´ (´My Voice´), ´La palabra´ (´The Word´), ´Partida´ (´Departure´), ´X´ (´X´), ´Circuito´ (´Circuit´) and ´Ya es tarde´ (´Already Late´) [69].

**XX century. Text in prose.** We will now choose the writer Miguel Delibes Setién (1920–2010) [70] [71] in his work ´La sombra del ciprés es alargada´ (´The Shadow of the Cypress is Lengthened´), from 1948, from which we will select the first chapter [72].

**XX century. Text in prose.** Finally, as we did in the case of Latin, we are going to reconsider the pericopes of St. Luke's Gospel which narrate the Presentation of the Lord in the Temple (Lk 2,21–39) along with the previous scenes of the Annunciation to Mary by the Angel Gabriel (Lk 1,26–38), the Visitation of the Virgin Mary to her cousin Elizabeth (Lk 1,39–56) and the Birth of Christ in Bethlehem (Lk 2,1–20). It is a text in prose from the edition of the Bible by Fr. Alberto Colunga Cueto, O.P. (1879–1962) and Eloíno Nácar Fúster (1870–1960), the Nácar-Colunga Bible, of the year 1961, a translation into Spanish from the original Greek text by Luke very adjusted to the original sense of the sacred redactor [73].

#### Graphemic-phonetic correspondences

The set of vowels in cases where they are accented with an accent has been reduced to their characters without the suprasegmental sign, which does not imply a change in sound and does not provide differential aspects in our analysis. This occurs in the vowels {<á>, <é>, <í>, <ó>, <ú>} and in the consonant <ý>. We also have the case of {<ï>, <ü>}, which have a diacritical sense of either a diphthong rupture or a phonetic mark, which we will group together in our phonetic transcription, however this diaeresis will be eliminated in the analysis of the graphemes.

Thus, the set of characters and phonological elements, in their correspondence, from the previous texts, where we notice that <k> and <w> do not appear, is shown in Table 5 [74] [75]:

**Table 5:** Spanish language. Latin alphabet (lowercase letters) and phonetic transcription (IPA symbols).

LATIN ALPHABETIC CHARACTERS (LOWERCASE)																									
IPA PHONETIC TRANSCRIPTION																									
**A**	**b**	**c**	**d**	**e**	**f**	**g**	**h**	**i**	**j**	**l**	**m**	**n**	**ñ**	**o**	**p**	**q**	**r**	**s**	**t**	**u**	**v**	**x**	**y**	**z**	**ç**
**A**	**β**	**k/θ/tʃ**	**d**	**e**	**f**	**g/x/ŋ**	**∄/tʃ**	**i**	**ʒ**	**l/ʎ**	**m**	**n/ŋ**	**ɲ**	**o**	**p**	**k**	**r/ɾ**	**s**	**t**	**u/w/∄**	**β**	**ʃ**	**i**	**dz**	**ts**

NOTE: The consonant <c> preceding <e> or <i> takes the sound /ɵ/. In the remaining cases, it is pronounced /k/. The set <ch> adopts the /tʃ/ sound. The graphemic group <gü> is pronounced /gu/. The <gue> and <gui> blocks are pronounced /ge/ and /gi/, respectively. For the rest <gu> remains as /gw/. The same consonant <g> preceding <e> or <i> remains as /x/. In all other cases the sound is /g/. The set <ll> changes to /ʎ/. On other occasions the consonant <l> becomes /l/. The <ng> group changes to /ŋ/. In all other cases <n> takes the phoneme /n/. The grapheme <q> appears linked to the vowel <u>, where the <qu> set preceding <e> or <i> remains as /k/; before <a> as /ku/; it does not exist before <o>; and finally, in the rest of the cases <qu> turns into /ku/, and there are no other possible situations than those reviewed here for the consonant <q>. The <rr> set and <r> at the beginning of the word takes the sound /r/. Between vowels and following <b>, <t> or <p>, the grapheme <r> changes to /ɾ/. In all other cases <r> takes the phoneme /r/.

## Persistence in time of a language

We do not intend to deal with variations in time of languages in this study as it is a very complex subject and with so many aspects to be taken into account beyond those we can collect and analyze here. However, not even some of them or even a single language in particular, is rich and complex enough, something that is outside the aim of this paper [76]. However, we will make some notes on various aspects referring to some of the tools used in Cryptology, such as the Frequency and the Index of Coincidence, applied to both graphemes and phonemes.

### Latin

#### Graphemic units

**Fig 2:**
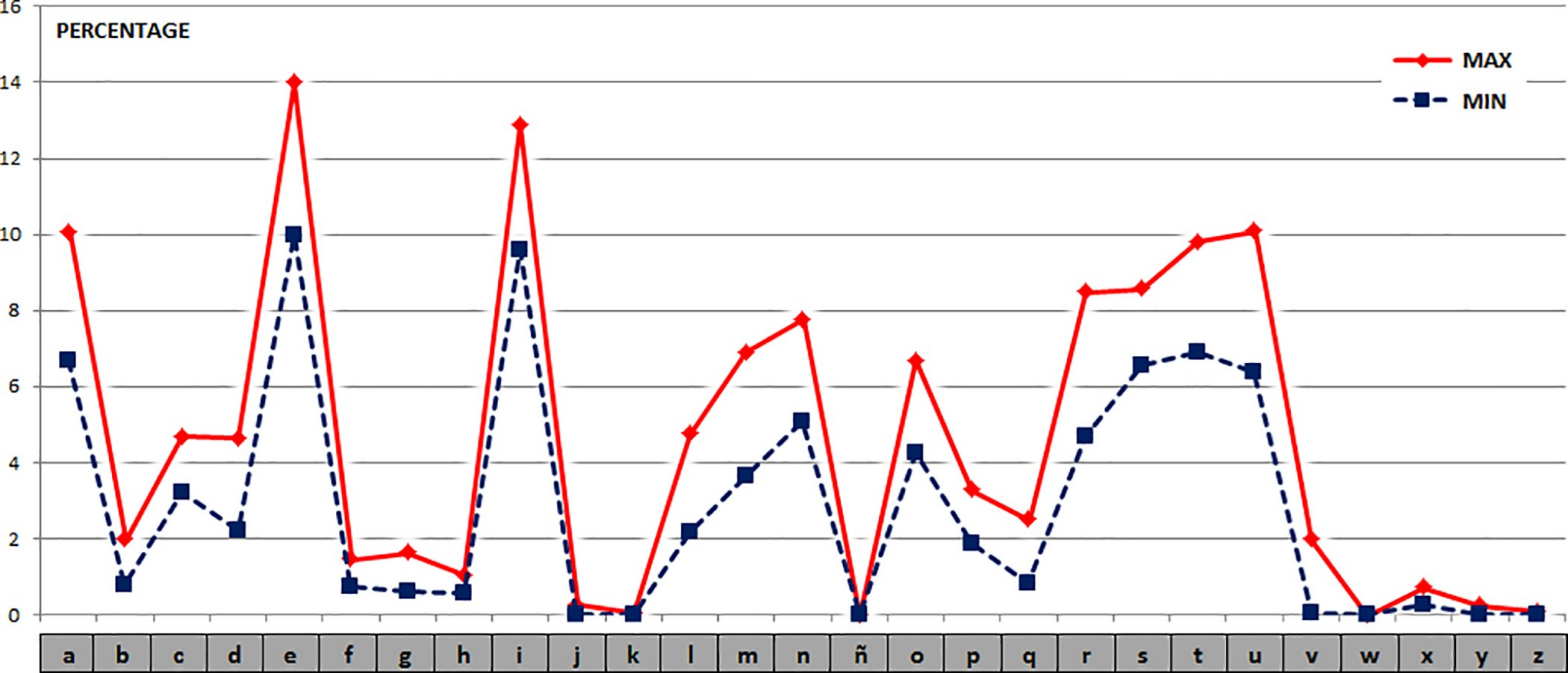
Frequency values of the graphemic units of the 10 Latin texts.

**Frequency Distance** For the set of Latin graphemic units, the percentages of the maximum and minimum values obtained for the selected texts are shown in Fig 2. Distances (in percentage values) between the graphemic units of all Latin texts versus the first one (which is used as reference) show the following values: {18.4, 21.5, 15.3, 10.1, 22,0 14.1, 12.6, 20.3, 12.0}.

It is possible to see a certain likeness between the different texts, in spite of the diacrony. Now if we compute the Manhattan distances (L_1_) of the 10 texts paired together, we can obtain the following 45 results (ordered from lowest to highest): {8.5, 10.1, 10.8, 11.3, 11.8, 12.0, 12.5, 12.6, 13.3, 13.3, 13.4, 14.1, 14.1, 14.1, 14.5, 14.7, 15.1, 15.3, 15.3, 15.8, 16.2, 16.2, 16.5, 16.6, 16.7, 16.8, 17.2, 17.5, 18.1, 18.2, 18.3, 18.4, 18.4, 18.4, 18.5, 18.9, 20.0, 20.3, 20.3, 21.5, 22.0, 22.7, 24.6, 26.6, 27.1}.

We can obtain a series of data for these 45 values:

The Mean μ = $\frac{1}{n}\sum_{i=1}^{n}x_{i}$ = 16.3.

The Mean Deviation D_m_ = $\frac{1}{n}\sum_{i=1}^{n}\left|x_{i}-\bar{x}\right|$ = 3.4.

The Standard Deviation σ = $\sqrt{\frac{1}{n-1}\sum_{i=1}^{n}{\left(x_{i}-\bar{x}\right)}^{2}}$ = 4.7.

Now we will calculate the sum of the distances of each Latin text with all the others, as an indicator of the text which departs most from the rest. The values obtained are: {146.5, 138.0, 163.9, 178.2, 124.6, 171.4, 139.9, 128.4, 171.5, 138.1}.

It is clear that the most distant ones are the 4th, 6th and 9th texts, corresponding respectively to the texts in verse by Venantius Fortunatus ´Hymnus in Honore Sanctae Crucis´, ´De Ecclesia Parisiaca´ and ´Pange, Lingua´, all of the second half of the 6th century, on the one hand, as the 4th text; the anonymous songs of the 11th-12th centuries of the ´Carmina Burana´ for the 6th; and finally, as 9th text the fragments by Thomas Aquinas, O.P. of the ´Summa Theologiae´, written between 1266 and 1268. On the other hand, the 5th and 8th texts are the ones with the lowest total distance, corresponding respectively to the texts taken from the work ´De Medicina´, inserted in the ´Etymologiarum Libri Viginti Sive Origines´ by Isidorus Hispalensis, written between 616 and 636, and the sermon ´In Purificatione Beatae Mariae Virginis´ by Antonius Patavinus, O.F.M., of the 13th century.

**Fig 3:**
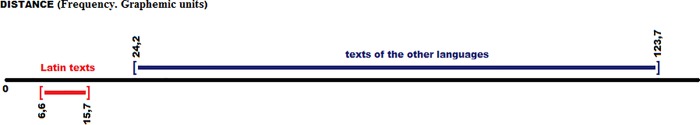
Distances between the Latin (average value) with all the texts in all the languages (graphemic units).

By comparing the average value of the Latin language with the rest of the texts, including those in their own language (a total of 261 texts; 10 in Latin), the results are shown in the Fig 3, highlighting the Latin texts in red versus the rest, in blue:

The 10 closest texts are the Latin ones, which correspond to the following texts: {Latin5, Latin8, Latin10, Latin7, Latin2, Latin1, Latin3, Latin9, Latin6, Latin4}. Following these, the nearest one in distance is quite far, with a value of 24.2, about 9 points farther than the previous value, the 4th Latin text, with a distance of 15.7. The values of the texts that follow the 10 best ones are: {24.2, 28.0, 28.5, 28.7, 28.9, 29.3, 29.3, 29.6, 30.2, 30.3}, corresponding to the languages and texts {Sardinian2, French-Provençal1, French3, Romanian1, French2, Tuscan2, French1, Scottish3, Sardinian1, Romanian3}, in general, but not Scottish, which is from the Germanic Family, all languages are from the Romance Family, Italic Subphylum.

The values of the texts farthest from the average value of Latin language are, for the 10 most distant cases, the values {110.9, 116.6, 118.8, 119.9, 120.7, 120.8, 121.5, 121.6, 122.0, 123.7} of a maximum value of 200, corresponding to the texts {Persian2, Persian1, Persian3, Arabic1, Hebrew2, Guanche2, Hebrew1, Punic1, Punic2, Arabic2}. However, we must not forget that several texts of greater value correspond to literary examples clearly or highly consonantal (Punic and Hebrew), which elevates the comparative distance.

**Index of Coincidence.** By analyzing the I.C. of our 10 texts, which does not take into account the order of the elements, and only the frequency of the graphemes, we have the following series of values: {0.0727, 0.0757, 0.0783, 0.0695, 0.0716, 0.0724, 0.0735, 0.0734, 0.0745, 0.0698}.

The Mean μ = 0.0731, the Mean Deviation Dm = 0.0019 and the Standard Deviation σ = 0.0026. These results indicate that in this case, the Index of Coincidence, compared to the Frequency distance, shows that the values are very close to the Mean value.

**Fig 4:**
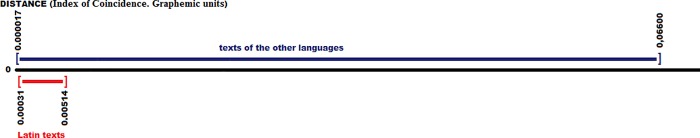
Index of Coincidence of the texts in Latin (graphemic units) (red) versus the rest of the texts (blue).

By observing the value of the I.C. differential of all the texts, we obtain the following graph, Fig 4, in which again we have highlighted in red the texts in Latin versus the rest ones, in blue.

We can see that the I.C. range value of the texts in Latin is broad enough to encompass a multitude of other languages and other texts, so it does not serve as a fine discriminant as it did in the case of the distances of the frequency of each graphemic unit, such as we have seen before. However, it is a result that helps to filter around 50% of the languages and their texts.

#### Phonetic units

**Fig 5:**
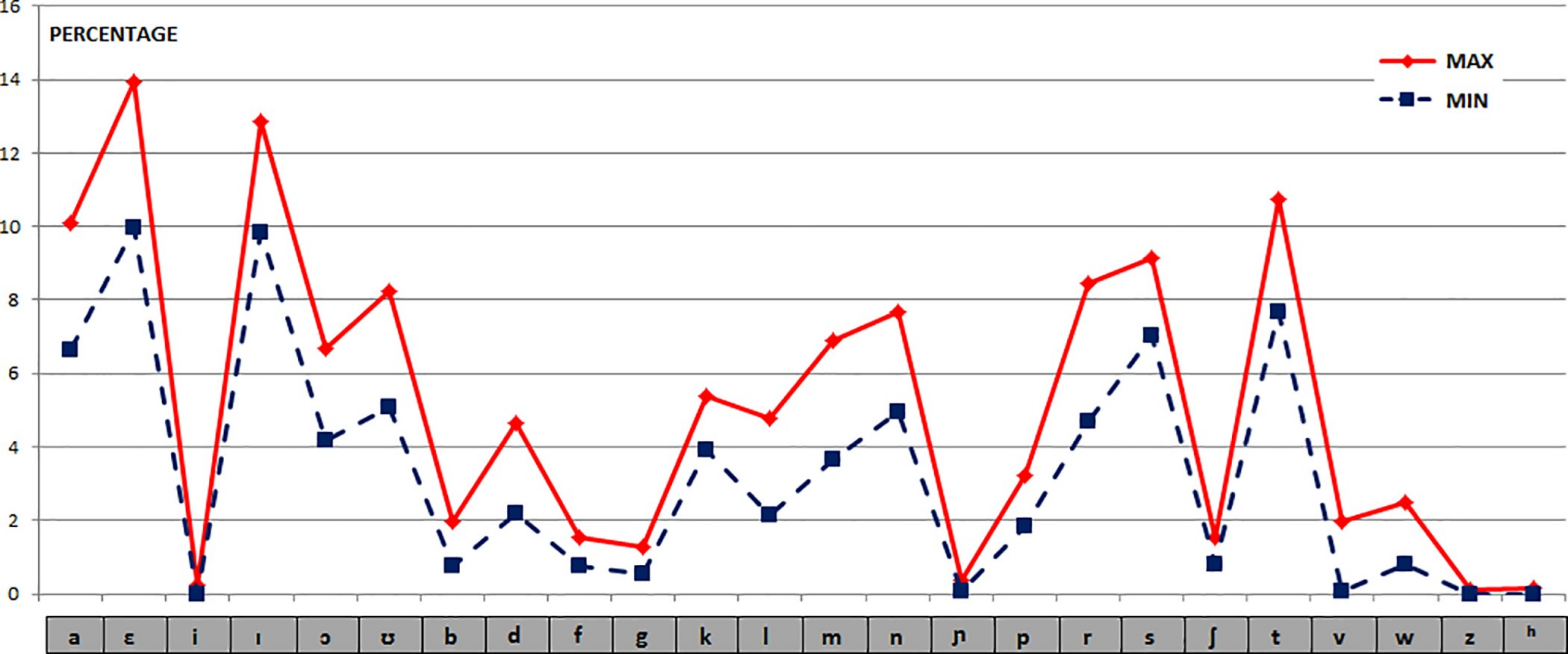
Frequency values of the phonetic units of the 10 Latin texts.

**Frequency Distance.** For the set of Latin phonetic units, the percentages of the maximum and minimum values obtained for the selected texts are shown in Fig 5. The distances (in percentage values) between the phonetic units of all the Latin texts versus the first one have the following values: {18.1, 21.5, 14.9, 9.8, 22.2, 13.9, 12.6, 19.9, 11.3}.

We can find a great similarity in this case again, clearly due to a very high correspondence between graphemic and phonetic units in the same language. By calculating all the Manhattan distances (L_1_) of the 10 texts paired together we have get (ordered from least to greatest): {8.4, 9.8, 11.1, 11.3, 11.5, 11.6, 12.5, 12.6, 13.1, 13.5, 13.7, 13.8, 13.9, 14.2, 14.3, 14.4, 14.9, 15.4, 15.5, 15.7, 15.8, 15.9, 16.1, 16.4, 17.0, 17.1, 17.5, 17.5, 17.6, 17.8, 17.8, 17.9, 18.1, 18.3, 18.3, 18.6, 18.7, 19.2, 19.9, 21.5, 22.2, 22.5, 23.6, 24.7, 25.2}.

For these data the Mean μ = 16.0, the Mean Deviation Dm = 3.2 and the Standard Deviation σ = 4.4.

The calculation for the phonetic distances to choose the furthest text from the rest is made using the same procedure applied for graphemic units, giving the following values: {144.4, 143.1, 162.0, 166.8, 122.2, 172.6, 139.5, 127.1, 166.7, 131.1}.

As we expected, and due to the high correspondence between letters and phonetic units in Latin, the results are almost similar to the graphemic case, the furthest being the 6th, 4th and 9th texts. On the other hand, as in the previous case, the 5th and 8th texts are the closest ones.

**Fig 6:**
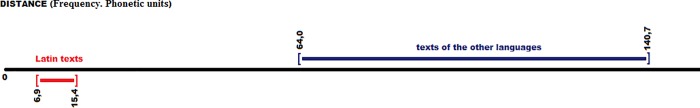
Distances between the Latin (average value) with all the texts of all the languages (phonetic units).

By comparing the average value of Latin with the rest of the texts, including those of their own language (a total of 261 texts; 10 in Latin), the results obtained are shown in the Fig 6, in which we have highlighted Latin texts again, versus the rest, colored in blue:

The 10 closest texts have the Latin values (in ascending order): {6.9, 7.4, 7.4, 9.1, 9.8, 10.7, 13.0, 13.2, 14.2, 15.4}, corresponding to the texts {Latin5, Latin10, Latin8, Latin7, Latin1, Latin2, Latin3, Latin9, Latin4, Latin6}. We can verify that the order is very similar to those of the previous one of the graphemic units. It follows them a distant text, with 64.0 of distance, about 50 points farther from the worst of the Latin texts. The values of the texts that follow the 10 best ones are {64.0, 64.9, 68.8, 70.8, 71.4, 72.1, 72.3, 72.8, 73.2, 76.4}, and correspond to the languages {LowGerman1, German2, Norwegian3, Albanian1, German3, Croatian2, Croatian3, Ukrainian2, Lithuanian1, Prussian3}, and as we can see, they are different from the values we have obtained in the analysis of the graphemic units, because of the non-absolute correspondence between graphemes and phonemes, since many graphemes in these languages that have been mentioned here in this phonetic section, do not exist in the previous graphemic analysis, all those within the scope of the Romance Family, with the exception of Scottish3, which was obtained in the 18th place.

The texts that show values furthest from the phonetic average Latin have a distance of {129.2, 129.7, 130.9, 131.1, 131.4, 132.0, 132.1, 133.3, 135.9, 140.8}, values even further than in the graphemic case, corresponding to the languages {Turkish2, Turkish3, Persian2, Nama3, Nama1, Finnish2, Persian3, Nama2, Kabyle1, Persian1}.

**Index of Coincidence.** Analyzing the I.C. for our 10 texts, which does not take into account the order of the elements, and only the frequency of the phonemes, we have the following values: {0.0720, 0.0765, 0.0781, 0.0704, 0.0719, 0.0724, 0.0733, 0.0739, 0.0739, 0.0697}.

The value of the Mean μ = 0.0732, the Mean Deviation Dm = 0.0019 and the Standard Deviation σ = 0.0025. We can check again that the values are very close to the Mean value μ in the case of the Index of Coincidence, compared to the distance in Frequency.

**Fig 7:**
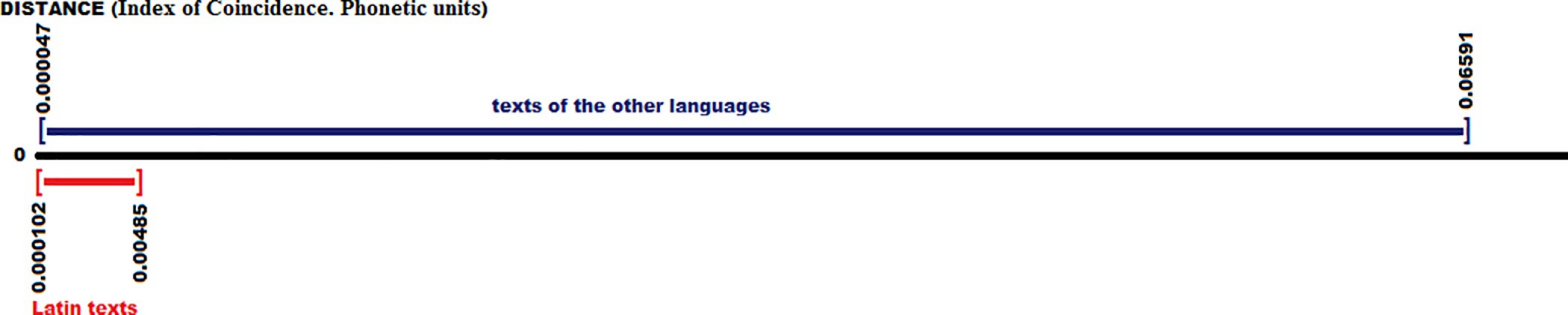
Index of Coincidence of the texts in Latin (phonetic units) (red color) versus the rest of the texts (blue color).

We show now in the Fig 7 the value of the I.C. differential in all texts:

It is clearly appreciable that the I.C. range of the values of the texts in Latin is broad enough to include a large number of other languages and texts, so it is not fit for purpose to make a fine discrimination between texts or languages, as opposed to distances in Frequency, although it manages to filter around 60% of texts and languages.

### Spanish (Castilian)

#### Graphemic units

**Fig 8:**
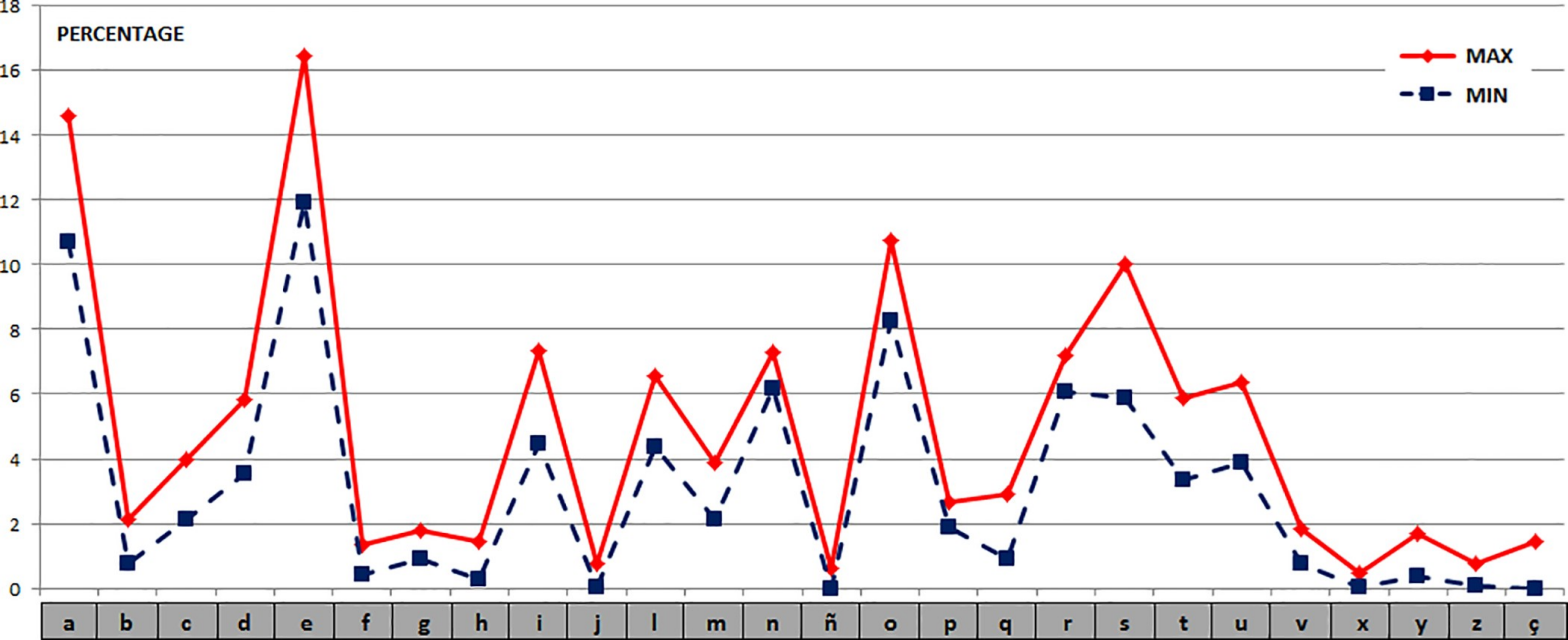
Frequency values of the graphemic units of the 10 Spanish or Castilian texts.

**Frequency Distance.** For the set of Spanish graphemic units, the percentages of the maximum and minimum values obtained for the selected texts are shown in Fig 8. The distances (in percentage values) between the graphemic units in all the Spanish texts versus the first one (which will be simply a reference) have the following values: {24.0, 16.3, 16.2, 19.2, 15.7, 15.3, 16.9, 16.0, 14.6}.

We can note a great resemblance between the different texts in spite of the passing of time. By calculating the Manhattan distances (L_1_) for all the pairs of texts, we obtain (ordered from lowest to highest): {10.1, 10.5, 11.0, 11.0, 11.1, 11.4, 11.8, 11.9, 12.4, 12.4, 12.9, 13.0, 13.1, 13.3, 13.3, 13.6, 13.9, 14.1, 14.1, 14.2, 14.6, 15.3, 15.7, 16.0, 16.2, 16.3, 16.5, 16.7, 16.9, 18.7, 19.2, 19.2, 19.5, 20.0, 20.4, 20.6, 20.9, 21.3, 21.9, 22.0, 22.6, 23.4, 24.0, 25.1, 25.6}.

For those 45 values μ = 16.0, Dm = 3.9 and σ = 4.9.

By analyzing the distance for each Castilian text with the rest, by a sum of distances, we have obtained the following values: {154.5, 201.0, 176.6, 137.5, 136.5, 126.5, 144.7, 131.8, 133.3, 136.2}.

The furthest texts are the 2nd and the 3rd ones, corresponding respectively to the literary work in prose by the Infante Juan Manuel in his ´Libro de los Enxiemplos del Conde Lucanor et de Patronio´, from around 1330; and the fragments in verse by Juan Ruiz, the Archpriest of Hita, of ´Libro del Buen Amor´, dated around 1330 or 1343. The closest one of them all is the 6th, which corresponds to ´El ingenioso hidalgo don Quixote de la Mancha´, from 1605, by Miguel de Cervantes Saavedra, very closely followed by the 8th and the 9th texts, corresponding respectively to ´Espadas como labios´ (1932), by Vicente Aleixandre y Merlo, and to ´La sombra del ciprés es alargada´ (1948), by Miguel Delibes Setién.

**Fig 9:**
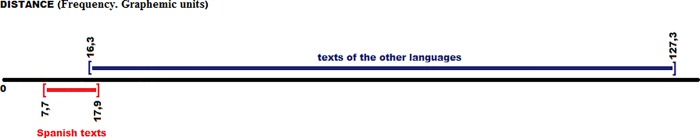
Distances between the Spanish or Castilian (average value) with all the texts of all the languages (graphemic units).

By comparing the average value of the Spanish language with the other texts, including those in their own language (a total of 261 texts; 10 in Spanish), the results obtained are shown in the chart below, Fig 9, always highlighted in red the Spanish texts versus the rest in blue:

The list of the values of the best 20 texts, the closest ones to the average Castilian or Spanish is as follows: {7.7, 8.4, 8.5, 8.9, 9.5, 9.8, 10.2, 12.4, 15.6, 16.3, 16.5, 16.9, 17.9, 21.0, 23.4, 23.7, 23.9, 25.3, 27.6, 28.0}. These distance values are those corresponding to the following texts: {Castilian6, Castilian4, Castilian5, Castilian8, Castilian9, Castilian10, Castilian7, Castilian1, Castilian3, Galician-Portuguese3, Balearic1, Galician-Portuguese1, Castilian2, Galician-Portuguese2, Lombard1, Catalan1, Ladino1, Venetian1, Sardinian2, French3}. Thus, the first nine are for Castilian, as the text in this language does not appear in the first ten corresponding to the Infante Juan Manuel, which goes to 13th place, although it is close in distance. We can see clearly that there is no a gap in distance as in the case of Latin, in which there was a clear difference between the Latin group and the rest of the languages; there is a certain ´continuum´ here, showing closeness in distance from Castilian with languages such as Galician-Portuguese, Catalan and Balearic, even French, or languages from Italy, both north and south, clearly close in distance to Castilian or Spanish.

The values of the texts farthest away from the average Spanish are, for the 10 most distant cases, {102.6, 110.4, 115.1, 119.2, 121.1, 121.6, 121.7, 124.0, 126.5, 127.3}, of a maximum of 200, which indicates a greater distance, and corresponding to the languages and texts {Hebrew1, Hebrew2, Punic1, Punic2, Persian2, Persian3, Persian1, Guanche2, Arabic1, Arabic2}. We can see that the largest distance in the case of Spanish corresponds to the same languages as in the case of Latin. Furthermore, as we have said before, we must not forget that several texts of a greater value correspond to literary examples clearly or highly consonantal, which increases the comparative distance.

**Index of Coincidence.** We have the following data by analyzing the I.C. for our 10 texts, which does not take into account the order of the elements, and only the frequency of the graphemes: {0.0759, 0.0765, 0.0737, 0.0762, 0.0767, 0.0751, 0.0750, 0.0735, 0.0755, 0.0743}.

The Mean μ = 0.0752, the Mean Deviation Dm = 0.0009 and the Standard Deviation σ = 0.0011. These data indicate that with the I.C., compared to the Frequency distance, the values are much closer to the Mean μ.

**Fig 10:**
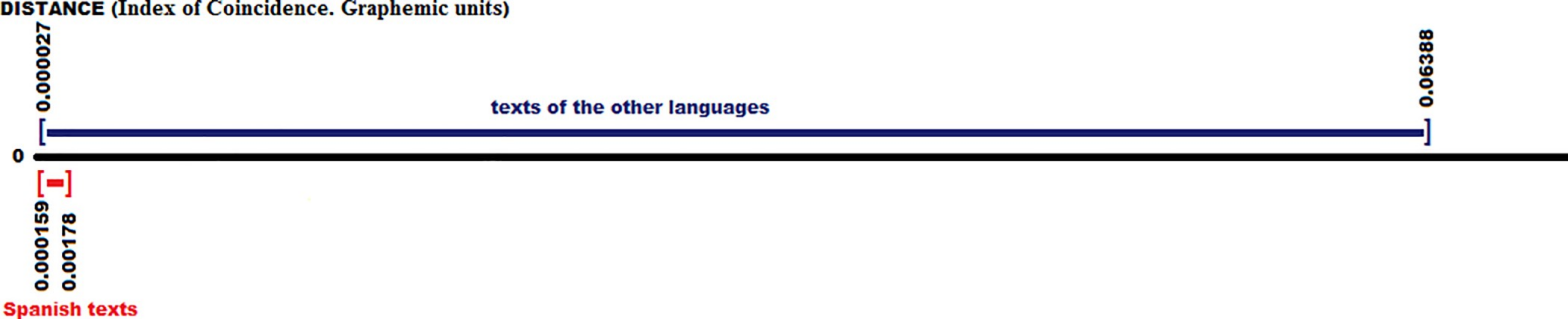
Index of Coincidence of the texts in Spanish or Castilian (graphemic units) (red) versus the other texts (blue).

The Fig 10 shows the distance with the average value or differential with the mean value of the Index of Coincidence in all texts, highlighting the texts in Spanish in red versus the others, in blue.

The I.C. of the texts in Castilian or Spanish is broad enough to encompass a large number of other languages and other texts, as happened with Latin, and therefore it is not useful as a discriminator and a fine differentiator, which did occur in the case of the analysis for the frequencies of appearance of the graphemic units and their distances, although it filters around the 70% of the languages and texts.

#### Phonetic units

**Fig 11:**
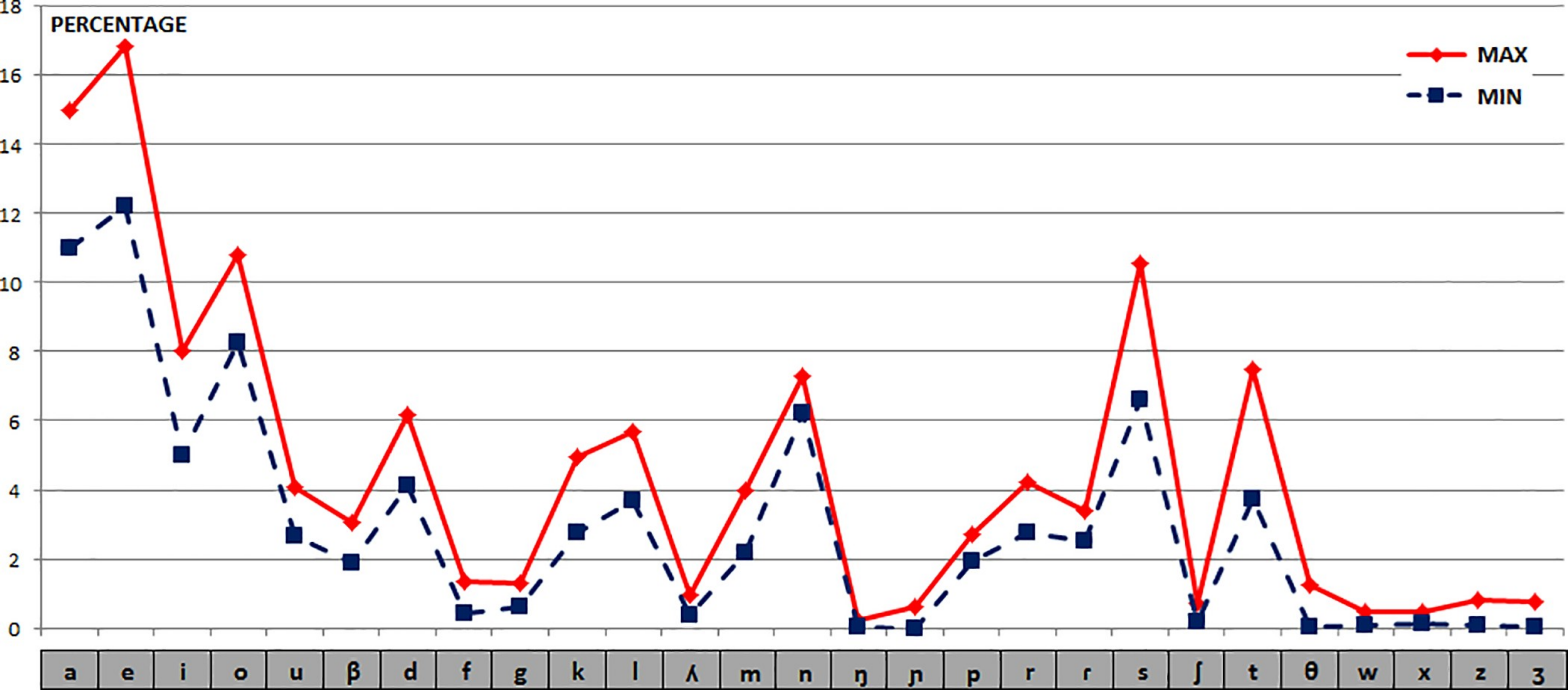
Frequency values of the phonetic units of the 10 Spanish or Castilian texts.

**Frequency Distance.** For the set of Spanish phonetic units, the percentages of the maximum and minimum values obtained for the selected texts are shown in Fig 11. The distances (in percentage values) between the phonetic units in all Spanish texts compared to the first one have the following values: {19.8, 15.0, 14.0, 15.2, 13.5, 13.3, 12.7, 12.7, 13.0}.

As we have seen before for Latin, and due to the strong correspondence between graphemes and phonemes, although there is a lesser degree of bijectivity in Castilian, we also have a similar relation of distances between texts in this case. If we calculate the Manhattan distances (L_1_) for the 10 texts paired together we get the following 45 results (ordered from lowest to highest): {8.5, 8.7, 9.6, 10.4, 10.7, 11.5, 11.9, 11.9, 12.0, 12.1, 12.2, 12.2, 12.4, 12.7, 12.7, 13.0, 13.1, 13.3, 13.5, 13.7, 13.9, 14.0, 14.5, 14.8, 15.0, 15.2, 15.3, 15.6, 15.7, 16.7, 16.7, 17.2, 17.7, 17.8, 17.9, 18.3, 18.8, 19.1, 19.3, 19.3, 19.6, 19.7, 19.8, 22.9, 23.0}.

For these 45 values: μ = 14.7, Dm = 3.1 and σ = 4.1.

Then, by calculating the Frequency distance for each Castilian text in its phonetic units with the others as the sum of all distances to them, we get the following data: {129.6, 175.7, 158.5, 132.2, 128.1, 117.4, 142.4, 112.9, 117.4, 137.0}.

The most distant texts are the 2nd and 3rd ones, just as in the graphemic case. The nearest ones are the 8th, 6th and 9th, very similar to the case of the graphemic units.

**Fig 12:**
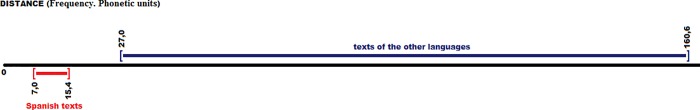
Distances between the Spanish or Castilian (average value) with all the texts of all the languages (phonetic units).

If you then compare the average value of Spanish with all the texts (261 texts; 10 in Spanish), the values obtained are shown in Fig 12, again marking the Spanish texts in red versus the others, in blue:

The 20 best of all the values are: {7.0, 7.1, 7.2, 8.4, 8.8, 9.1, 10.4, 11.4, 13.4, 15.4, 27.0, 28.4, 29.2, 30.8, 35.4, 36.1, 37.5, 38.2, 39.5, 40.5}, corresponding to the texts {Castilian6, Castilian8, Castilian9, Castilian5, Castilian4, Castilian1, Castilian10, Castilian7, Castilian3, Castilian2, Galician-Portuguese3, Galician-Portuguese1, Ladino1, Galician-Portuguese2, Romani4, Tuscan2, Tuscan1, Balearic1, Venetian2, Venetian1}. As we can see, here the best 10 texts are all of the Castilian ones. In addition, the order is quite close to that of the graphemic units. We can also observe that there is a certain difference between the Castilian group and the rest of the following texts in distance, having a gap of about 12 units with the 11th text, something that did not occur in the case of the graphemic units. The set of the texts between the 11th and the 20th is largely similar to those found before, coinciding in that way Galician-Portuguese, Balearic, Ladino and Venetian, appearing here Tuscan and Romani, however there was neither Catalan nor French, which had been there, due to the differences between graphemes and phonemes. In any case, there is sufficient coherence, with their own dissimilarities, between graphemes and phonemes.

The 10 most distant texts from the average Spanish are those with the values {127.0, 127.7, 128.1, 129.0, 129.4, 139.2, 144.0, 154.8, 158.8, 160.6}, corresponding to the texts and languages {Kabyle2, Kabyle3, Kabyle1, Abkhaz2, Abkhaz1, Hebrew1, Hebrew2, Punic2, Breton1, Breton2}, respectively, of a maximum of 200 units in distance, which indicates a very high remoteness. We must also emphasize here that only in the case of Hebrew and Punic do we find equality in this group with the results that we have obtained for the graphemic case, explained by the differences between graphemes and phonemes.

**Index of Coincidence.** We have the following values by analyzing the I.C. for our 10 Spanish texts, which does not take into account the order of the elements, and only the frequency of the phonemes: {0.0763, 0.0788, 0.0757, 0.0773, 0.0782, 0.0768, 0.0764, 0.0743, 0.0768, 0.0759}.

The Mean μ = 0.0767, the Mean Deviation Dm = 0.0009 and the Standard Deviation σ = 0.0012. Also in this case we can observe that the Index of Coincidence, compared to the Frequency distance, has its values very close to the Mean value.

**Fig 13:**
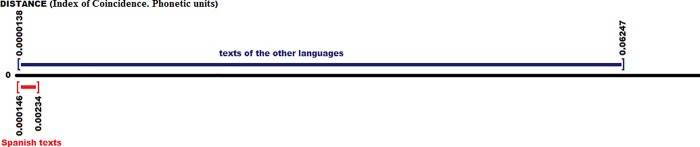
Index of Coincidence of the texts in Spanish or Castilian (phonetic units) (red) versus the rest of the texts (blue).

We show the I.C. differential value of all texts in Fig 13:

We can clearly see that the I.C. of the texts in Spanish or Castilian is broad enough to include a multitude of other languages and other texts, although it manages to filter about 70% of languages and texts, far from their values. Once again, this is the reason for not considering this variable as a fine discriminator when it comes to filtering texts and languages, as was the case with the study of the Frequency distance.

### The rest of the languages

After the intra and inter linguistic analysis for Latin and Spanish, we will try to corroborate this persistence over time with the remaining languages analyzed. We will do so only in the case of frequencies, both graphemic and phonetic units, since the Index of Coincidence is less discriminatory. Although, in these other cases the selection of texts has been lower than in Latin and Spanish, as the diachronic strength has been observed in the texts of both languages. However, and due to the temporal amplitude and the wide range that has been taken, they will serve to give us an appropiate idea of the diachronic maintenance or not in a large number of languages in diverse linguistic phyla.

If we take the average value from each language in its graphemes (and in its phonemes) separately, and we calculate the Frequency distance with the remaining the texts in the same language, the greater value from these data will be our reference; if that value is compared with the other languages and their texts, the texts that are below this maximum value, and therefore could be as a text of such a language, are are of only fifteen languages, which we will consider next, leaving the rest perfectly clear and distinguishable against other languages:

#### Galician-Portuguese

Within the graphemic range of Galician-Portuguese are included the texts Castilian4 and Castilian5, surpassing the maximum value of Galician-Portuguese, that is 14.4. Both languages, Spanish (Castilian) and Galician-Portuguese, belong to the Romance Family, Western Subfamily, Galo-Iberian-Romance Group and Ibero-Romance Subgroup. It is a low value, explained by the proximity between these languages, more when it is due to a text in verse of the 13th century (ca. 1269–1280), as they are two Cantigas by Alfonso X the Wise (1221–1284), Galician-Portuguese1 [77], versus Castilian4, the text in verse ´Las coplas por la muerte de su padre´ (´The Couplets on the Death of his Father´) (ca. 1476), by Jorge Manrique Toletanus (1440–1479), and the Castilian5 text, a fragment in prose of the ´Libro de Calixto y Melibea y de la Puta Vieja Celestina´ (´Book of Calixto and Melibea and the Old Whore Celestina´) (1499), by Fernando de Rojas (ca. 1476–1541). The other two texts of the Galician-Portuguese that lead to such a differential distance are Galician-Portuguese2, the fragment of ´Da Batalla de Rroçauales et da Morte de Rrulã et dos outros lidadores´ (´The Battle of Roncesvalles and the Death of Rulã and other Knights´), of the end of 14th century or beginnings of the 15th, a work written by an unknown author [78]; and Galician-Portuguese3, a text in prose of the ´Libro do Concello de Santiago´ (´The Book of the Council of Santiago´) (1416–1422), by an anonymous autor, selection of an account of the events of the year 1417 [79].

#### Spanish

Within the Castilian range of the graphemic units we can find the texts Galician-Portuguese1, Galician-Portuguese3 and Balearic1, surpassing the maximum value of Castilian, which is 17.9. The reasons are easily explained again because of the texts considered, all of them of languages of the Romance Family, Western Subfamily, Galo-Iberian-Romance Group and Ibero-Romance Subgroup, and the time of their writing. Thus, the maximum Spanish value corresponds to Castilian2, a text in verse of ´Libro del Buen Amor´ (´The Book of Good Love´), dated around 1330 or 1343, by Juan Ruiz, Archpriest of Hita (died ca.1351). Galician-Portuguese1 and Galician-Portuguese3 have been mentioned previously, the Balearic1 text being a fragment of ´Blanquerna´ (´Blanquerna´), of the year 1283, by the franciscan friar Raimon Llull, T.O.R. (1235–1315) [80].

#### French-Provençal

Within the French-Provençal graphemic range are included Balearic1, French1, French2 and French3 texts, surpassing the maximum value of French-Provençal, which is 21.8. The proximity between these languages (especially with French), but all of them of the Galo-Iberian-Romance Group, explains this overlap, even more considering the temporal amplitude of the texts, French-Provençal2 being the text that maximizes the distance, which corresponds to a few verses of ´La Piedmontoize en vers Bressan´ (´Piedmontese in Verse of Bressan´), dated on 1619, by Bernardin Uchard (1575–1624) [81]. The other texts in French-Provençal are French-Provençal1, text in prose by Marguerite d'Oingt (ca. 1240–1310), ´Speculum´ (´Mirror´), around 1294 [82]; and French-Provençal3, by Jean-Baptiste Cerlogne (1826–1910), ´La Bataille di Vatse a Vertosan´ (´The Battle of Cows of Vertosan´), of the year 1858 [83]. We have already spoken about the Balearic1 text, French1 being a set of verses of the poem ´Li tornoiemenz Antecrit´ (´The Antichrist's Tournament´), a work between 1234 and 1240, by Huon de Méry, author who can be placed around the first half of the 13th century [84]. On the other hand, French2 is a text in prose by Jehan Froissart (ca. 1337-ca.1405), of his ´Chroniques´ (´Chronicles´), which covers events from the year 1327 to 1400 [85]. The French3 text is a text in verse by the poet François Villon (ca. 1431- ca.1463), in particular of his work ´Grant Testament´ (´The Great Testament´), written in 1461 [86].

#### Scottish-Gaelic

Within the range of the graphemic units of Scottish-Gaelic are included the Irish-Gaelic2 and Irish-Gaelic3 texts, surpassing the maximum value of the Scottish-Gaelic, a high value of 32.8. Gaelic influence gives rise to this overlap, with such a value of distance, marked by the Scottish-Gaelic2 text, a work in verse of the first half of the 16th century, ´Leabhar Deathan Lios Mòir´ (´The Book of the Dean of Lios Mòir´), written between 1512 and 1542, and in the case selected, compiled by the bard Allan Mac Royre (15th-16th centuries) [87]. On the other hand, Scottish-Gaelic1 corresponds to a document of 1408, a charter of rights, ´Brontanas Mhic Dhomhuill nan Eilein´ (´Letter of Rights of Mhic Dhomhuill, Lord of the Islands´), by Mhic Dhomhuill (XIV-1423) to Bhrian Bhicaire Mhagaodh (14th-15th centuries) [88] [89]. As for Scottish-Gaelic3, it is a selection of Marian texts of the St. Luke's Gospel in the version of Bible from 1807 by the Scottish Promoting Society for the Diffusion of Christianity, ´Am Bìoball Gàidhlig´ (´Scottish Gaelic Bible´) [90]. On the other hand, Irish-Gaelic2 corresponds to a text in verse by the bard Gofraidh Fionn Ó Dálaigh (1320–1387), the poem ´A chros thall ar an dtulaigh´ (´Oh, Cross, up there on the hill´), from the end of the 14th century, but we are not sure of the exact date [91]. The Irish-Gaelic3 text is in verse, by the bard Tadhg Óg Ó hUiginn (died 1448), the poem ´Cia do-ghéabhainn Go Gráinne´ (´Whom could I send Gráinne?´), not much before the year 1440 [92].

#### Icelandic

Within the graphemic margin of the Icelandic enters the Norwegian2 text, surpassing the maximum value of the Icelandic language, a high value of 29.9. The text that maximizes the distance from the rest of the selection is Icelandic2, a work in verse of the Edda poetry, the ´Völuspá´ (´Prophecy of the Sibyl´), of uncertain date and anonymous authorship, and which we will take from the ´Codex Regious. GKS 2365 4to´ (ca. 1270) [93], a value that places in its range Norwegian2, a text in prose of the ´Saga of Þiðrek´, ´Þiðrekssaga´, a work from the 13th century of unknown authorship, relative to Dietrich von Bern, a name that in the northern half of Europe refers to Theodericus Magnus (454–526) for the Romans, king of the Ostrogoths [94]. The other two Icelandic texts considered are Icelandic1, the ´Ynglinga Saga´ (´Saga of the Ynglinga´), from the ´Snorra Edda´ (´Snorri's Eddas´), circa 1225 to 1250 by Snorri Sturluson (ca. 1178–1241) [95]; and Icelandic3, a set of texts of the St. Luke's Gospel of the ´Nýja Testamenti´ (´New Testament´) of the year 1540, translated by Oddur Gottskálksson (ca.1515-1556) [96].

The result of overlap of the graphemes is also found in the analysis of distances in the phonemes, where the text that remains within the range of the Icelandic is again Norwegian2, surpassing the maximum value of the Icelandic, a high value again of 25.5. Note that both Icelandic and Norwegian are languages from the Germanic Family, Northern Subfamily and Scandinavian Group, and during the 12th and 13th centuries the languages that we can differentiate today for Icelandic, Danish, Swedish or Norwegian were still joined, which would explain this overlap.

#### Norwegian

If we now consider the Norwegian language, within its range of graphemes includes the texts Icelandic1, Icelandic3, Danish2 and Danish3, it surpasses the maximum value of Norwegian, a high value of 34.6. The text in this language with such a high differential is Norwegian3, a text in prose by Absalon Pedersson Beyer (1528–1575) of his work ´Oration om M: Geble´ (´Prayer for Master Geble´), dated on 1571 [97]. The other texts analyzed in Norwegian language are Norwegian1, a text in prose from about 1250, the ´Konungs Skuggsjá´ (´Royal Mirror´), by an anonymous author [98]; and Norwegian2, just mentioned in the preceding study. The Icelandic texts that have entered in their range are the aforementioned Icelandic1 and Icelandic3. On the other hand the Danish texts are Danish2, a text in verse by Hans Christensen Sthen (1544–1610), in particular of his hymn work ´En Liden Haandbog´ (´Little Manual´), from 1578, his ´Dialogus eller Samtale imellem it Menniske oc Døden´ (´Dialogue between Man and Death´) [99]; and Danish3, a text in verse by Anders Sørensen Vedels (1542–1616), the ballad ´Den anden Vise om Herr Marsk Stig´ (´The Second Ballad of Mr. Marsk Stig´), summarized in 1591 [100]. The analysis of the phonemes shows that within the Norwegian band only the Icelandic1 text enters, defining the membership better than in the graphemic case, but surpassing the maximum value of Norwegian with this text, still a high value of 31.6. As mentioned above, these languages, not only Icelandic and Norwegian, but also Danish, belong to the same Group, Scandinavian, and until relatively few centuries ago have had a very similar mixed and united history, since the independence of Norway from Denmark in 1814 when there was to be a renaissance of Norwegian as its own distinct language.

#### German

The only text that falls within the German graphemic range is LowGerman1, surpassing the maximum value of that language, a value of 19.9, not too high, and which explains that both languages, which are from the Germanic Family, Western Subfamily and Continental Group, present similarities, even more at times when both languages were being defined. The text marking the maximum distance difference is German2, a text by Oswald von Wolkenstein (ca. 1376–1445), the lyric piece ´Ain burger und ain hofman´ (´The Bourgeois and the Courtier´), from around 1425 [101]. The other texts in German that mark the differences with the first text are, on the one hand, German1, which corresponds to ´Alexius´ (´Alexius´), which we can locate in the 13th century, of Wirzeburc ich Kuonrât (ca. 1225–1287) [102]; German3 being a series of fragments from St. Luke's Gospel of the translation of the Bible by Martin Luther (1483–1546) of the year 1545, ´Biblia Das ist, Die gantze heilige Schrifft, Deudsch´ (´The Bible, that is, the Complete Sacred Writings in German´) [103]. For its part, LowGerman1 is a text in verse, ´Sachsenspiegel´ (´The Mirror of Saxony´), from around 1220–1232, a legal book by Eike von Repgow (ca. 1180-ca. 1232) [104].

#### Low German

The texts Dutch1 and Dutch2 are found within the range of the graphemic units of Low German, surpassing the maximum value of that language, 19.5. LowGerman1 is the text which gives the aforementioned maximum value in distance. Both Low German and Dutch are languages from the Germanic Family, Western Subfamily, Continental Group and Lower Subgroup, hence their kinship, especially in the centuries of their conformation. The other works in Low German are a fragment in prose of ´Der grosse Seelentrost´ (´Great Relief for the Soul´), from about the middle of the 14th century, by an unknown author, which we refer as LowGerman2 [105]. LowGerman3 are some fragments in verse of ´Reynke de Vos´ (´The Fox Reynke´), from 1498, which is a translation to the Low German made in that year of the Dutch work of 1487 by Hinrek van Alkmaar (c. XV) [106]. For its part, the Dutch texts correspond to Dutch1, which is a text in verse, of unclear authorship, which we can date from around 1374, ´Beatrijs´ (´Beatrijs´) [107]; on the other hand, Dutch2 is another text in verse by the poet Anthonis de Roovere (ca. 1430–1482), fragment of ´Lof vanden heyjligen Sacramente´ (´Praised be the Holy Sacrament´), dated from around 1456 [108].

#### Albanian

Within the graphemic range of this language are the texts French1, German3, Dutch2, English1, English3, Frisian1, Frisian2 and Frisian3, surpassing the maximum value of Albanian, a very high 44.1, an overlapping that does not occur in their phonetic units, resolving this apparent resemblance in graphemes. The texts taken from the Albanian have so many differences that they distort the values, hence overlapping languages of families outside the Albanian Family, such as the Romance Family or the Germanic Family. This is because, on the one hand, we have taken one of the oldest texts ever, which we call Albanian1, a small rudimentary glossary with 46 terms, dated 1496, with some names, numbers and phrases in Albanian written by a German merchant, Arnold von Harfit (1471–1505) on a trip to Albania [109] [110]; Albanian2 is a fragment of a ´Meshari´ (´Missal´), by Gjon Buzukut (c. XVI), dated 1555 [111]; Albanian3, a mixture of prose and verse, is a series of fragments of ´E mbsuame e krështerë´ or ´Christian Doctrine´, of the year 1592, a Catechism by the Orthodox priest Lekë Matrënga (1567–1619) [112] from the doctrine book by the Jesuit Diego de Ledesma, S.I., ´Docttrina Christiana´ (1572).

#### Romani

Within the range of the graphemic units of Romani, we can find the texts Tuscan1, Tuscan2, Lombardo1, Venetian2, Napolitan1, Ladino1, Galician-Portuguese1, Castilian1, Castilian3, Castilian5, Castilian6, Castilian7, Castilian8, Castilian9, Castilian10, Croatian2, Swedish1 and Wolof1, surpassing the maximum value of Romani, a very high result of 39.1. These results are due to the discordance of the average of Romani language with the Romani1 text, a vocabulary taken from a list, the ´Winchester Confessions´, a confession made by a so-called Walter Hindes (c. XVI-XVII), a Londoner who learned the language through contact with Romani gipsy groups in London, when he was arrested in Winchester prison in 1616 [113], which even leads to overlap languages of the Indo-European Phylum with the Niger-Congo (Wolof) Phylum, something absurd. The analysis for the phonetic units concentrates data better, leaving only the texts Tuscan1, Tuscan2 and Ladino1 within the range of Romani, surpassing the maximum value of Romani, a still very high value of 37.2. Romani has had a very geographically dispersed literary history for centuries, and yet today, in diverse countries with its own and different languages, from the United Kingdom or Finland to Romania, Yugoslavia or Nepal, passing through Spain or France, highly dialectical and full of loaned words adapted to its own linguistics, at the same time as strongly traditional and oral. Romani has undergone a multitude of variations, changes and adaptations from its linguistic origins to the dissemination and nomadism of these peoples throughout Europe and part of Asia. In this case we have a text in written British Romani, and therefore, linked to the branch from the North of Europe (Polish, Russian, Scandinavian, Baltic, Germanic and Gallic areas), with all the pecularities that they may have versus other branches of the same language, such as the branch of Eastern and Slavic Europe (Czech, Slovak, Hungarian, Austrian and Balkan areas). However, due to its antiquity, we have taken it as the first text, with the exception of it being analyzed doubly: on the one hand, in the same way as it was written at that time in the confession, and on the other, as in the second text, Romani2, but this time in the spelling of the Welsh Romani as it was already articulated grammatically in the 20th century [113]. We can say that in this case, Romani2, data of graphemes/phonemes have a much better value of 34.5/31.2, respectively.

On the other hand, Romani3 is a tale from Romanian areas of Hungary, ´O Rom taj o Beng´ (´The Romani and the Devil´) compiled in 1984 from its oral tradition [114]; Romani4 being the Marian pericopes of St. Luke, already analyzed in other languages, of the New Testament in translation of the Jesus Army Church in 1984, ´E Lashi Viasta´ (´New Testament´) [115].

#### Awdjila

In this language the texts that are within its graphemic range are Tashelhiyt1, Tashelhiyt2, Djerba3, Ghadames2 and Tagargrent3, surppasing the maximum value of this language, a very high result of 49.2. The very high value for this language, which means that other languages of the Berber Family remain within its range of frequency distances, is due to the use of a first text, Awdjila1, the oldest written compilation, between the years 1932–1933 by Fernando Zanon (died c. XX), only six verses, which imply this discordance, furthermore, the other two texts are transliterations of the same hand [116]. Thus, Awdjila2 and Awdjila3 are short stories, ´Abú-dabăr u ámz̩a´ (´Abú-dabăr and the Ogre´) and ´Sîdi H̩ámed ĕz-Zarrûq´ (´Mr. H̩ámed ĕz-Zarrûq´), both collected by Umberto Paradisi (1878–1933) between 1959 and 1960 [117]. In the case of phonemes there is also overlap since within the band of Awdjila enter the texts Tashelhiyt1, Tashelhiyt2, Ghadames2, Tagargrent1, Tagargrent2, Tagargrent3, Figuig1, Tarifiyt2, Tarifiyt3, Chaouia1, surpassing the maximum value of Awdjila, a very high 48.7, although they are texts in the nearby Berber languages, from the Eastern and Northern Subfamily.

#### Tagargrent

The texts that are within the range of the graphemic units of the frequency distance of the Tagargrent are a great multitude, a total of 53, even from several phyla, because of the high maximum distance between the texts used, with a value of 50.6, so this information is not relevant, as explained by Tagargrent3, a text in prose, a report on life at home taken from inhabitants of the zone of Ouargla (Algeria), collected by Jean Delheure (1911–2001) and Maurice Jardon (died 1956) between 1941 and 1961 [118]. Its transliteration is very different in graphemes (and phonemes) with the other two texts selected from this language. Thus, Tagargrent1 is a text in verse that includes several songs collected between 1885 and 1887 by René Basset (1855–1924) [119]; and Tagargrent2 is a tale collected in 1906, ´Tanfoust n baba Brahim´ (´Tale of father Brahim´), by Samuel Biarnay (1879–1918) [120]. There is also overlap in the case of phonemes, although much lower than in the graphemic case, because within the Tagargrent range enters the texts Awdjila2, Tarifiyt1 and Tarifiyt3, this time only Berber languages, surpassing the maximum value of the phonetic Tagargrent, a high data of 40.3.

#### Tarifiyt

In the Tarifiyt language, although only in the analysis of the phonemes, since there is no graphemic overlap, within the range of this language we only have Chaouia1, surpassing the maximum value of Tarifiyt, a frequency distance of 31.4, due to the text Tarifiyt1. Both Tarifiyt and Chaouia are very close languages, both from the Berber Family, the Northern Subfamily, the Zenati Group and Riff Subgroup, which can explain this linguistic closeness. The text Tarifiyt1 corresponds to some verses of diverse songs compiled during 1965–1966 in the zone of the mountains of the Moroccan Riff by Terri Brint Joseph (1940–2002) [121]. In addition, Tarifiyt2 is a serie of habitual phrases and proverbs along with different names of parts of the human body, taken from the compilation by Mohamed Tilmatine (1956-), Abdelghani El Molghy (1964-), Carles Castellanos i Llorenç (1942-) and Hassan Banhakeia (1966-) published in 1998 [122] [123]; finally, Tarifiyt3 are fragments of the St. Luke's Gospel of a very recent version of the New Testament, published in 2009, ´Řexbar As̩ebh̩an n Yeccu Lmasih̩´ (´The Gospel of Jesus Christ´) [124]. For its part, Chaouia1 corresponds to a text in prose, a tale, ´Haqs̩it̠ n Lɣul d̠ t̠aqiyart̠´ (´Tale of the Ogre and the Beautiful Woman´), compiled by Gustave Mercier (1874–1953) at the end of the 19th century [125].

#### Oromo

The texts Kanuri1 and Kanuri3 enter within the phonetic frequency distances within the scope of the Oromo language, as there is no graphemic overlap, surpassing the maximum value of Oromo, 35.8. The Oromo1 text is a song in verse, a lullaby, together with two songs of caravans, compiled in the year 1922 by Enrico Cerulli (1898–1988) [126]. Likewise, Oromo2 is a set of proverbs compiled by the same Cerulli in 1922 [126]; and Oromo3 are fragments of St. Luke's Gospel of the version of the ´Holy Bible in Oromo´ (´Kitaaba Qulqulluu Orominya´), from 2006 [127]. The text that takes to this distance of value superior to 35 is Oromo3, due to the diversity of compilers of the texts, the first two by Enrico Cerulli at the beginning of the 20th century and the third one a revision of the phonemes being more updated and improved, although there was already a version of the Bible from 1899, this improved version in the language was made by the Bible Society of Ethiopia in 2006. Moreover, Oromo belongs to the Cushitic Family of the Afro-Asiatic Phylum, spoken by populations located in Ethiopia; Kanuri being from another Phylum, the Nilo-Saharan, and from the Saharan Family, a language spoken by the inhabitants of Nigeria, Chad, Cameroon or Niger. As we can see, peoples and very diverse ethnic groups to exist a true linguistic relationship between Oromo and Kanuri.

#### Kanuri

The texts Oromo1 and Wolof3 enters within the Kanuri range of graphemes, surpassing the maximum value of Kanuri, a high result of 29.3. In the study of phonemes also occurs superposition with Oromo1, surpassing the maximum value of the Kanuri, a value of 30.1. The text Kanuri1 corresponds to a series of proverbs compiled by the Protestant Reverend Sigismund Wilhelm Kölle (1820–1902) in 1854 [128]. Kanuri2 is a tale, also collected by S.W. Kölle himself and published in the same year 1854 [128]. In relation to Kanuri3, it is a text in verse, a hunting song, ´Kenzar´ (´Giraffe´), compiled by Thomas Geider (1952–2010) and Raimund Vogels (1956-) in 1995 [129]. The text in Kanuri that elevates the differential of frequency distances is Kanuri2, both in graphemes and in phonemes, possibly for having 452/446 graphemic/phonetic units compared to 2330/2321 and 3925/3877 of the texts Kanuri1 and Kanuri3, which leads to not reaching suitable frequency values, more when Kanuri2 was taken by the same compiler as Kanuri1. Note that Kanuri is a language from the Nilo-Saharan Phylum, while Oromo is from the Afro-Asiatic Phylum and Wolof belongs to the Niger-Congo Phylum, very distant to suppose any real linguistic relationship between them.

After an in-depth study for Latin and Spanish, as well as the subsequent analysis for the other languages, the nearness and similarity between their own graphemic and phonetic frequencies and indexes of Coincidence has been verified, in spite of the diversity of the languages considered. It is possible to affirm with the maintenance over time of the frequencial characteristics with considerable confidence that, as well as of the Index of Coincidence, of the graphemic and phonetic units of the languages, characteristics that can be used to identify them from the rest.

In a few languages we have found overlaps with other texts from other languages, although the use of literary works so disparate in time, even written during their linguistic conformation, sometimes very close to other languages, or different transliterated texts in high oral languages or the small number of units of the selected material, are some of the main explanations for the differences observed in our analysis, divergences that would be solved with a more meticulous observation in each particular case, but nevertheless they reaffirm the thesis of the diachronic invariance of the languages in their minimal graphemic and phonetic units and their use as differentiating patterns (in Frequency better than in I.C.) in contrast to the rest.

## Conclusions

Throughout history, cryptanalysts who faced a cipher text used to do it during periods of conflict, which allowed the languages used by the contenders to be known and thus the base language of the original text. What is more, the consideration of a particular language forced to study it in its synchronic properties, relegating a temporal and diacronic analysis, of little usefullness in those circumstances.

The underlying question that was pending was whether the two main tools of classic Cryptography, the analysis of the Frequency and the Index of Coincidence of their graphemes, remained in time as linguistic invariants and were able to be used as distinguishing elements in contrast to the rest of languages.

In order to do so we have analyzed a wide range of languages, a total of 101, from the geographical area of Western Eurasia and Africa, which includes the Indo-European, Uralic, Altaic, Caucasian, Afro-Asiatic, Nilo-Saharan, Niger-Congo, Khoisan and Austronesian phyla, as well as two unclassified languages, a total of 261 texts between the 6th century BC to the 21st century, examining their frequency characteristics and their Index of Coincidence of their graphemic units, which we have extended to the study of phonemes, a novel aspect [12].

Thus, beyond the already available cryptological knowledge of a couple of dozen European languages, we present results and conclusions of considerable linguistic scope, although with the desire to extend it in the future to Asian phyla and families which are not yet studied and those of the American continent and Oceania.

It was assumed by the cryptographers that different texts written in different languages, despite being encrypted, maintained some of their own patterns such as the Index of Coincidence and even the frequency of the elements, the letters. At least in about a dozen languages, mainly languages ​​of European origin, from the Indo-European Phylum. This assumption was due not so much to a systematic and rigorous study but to experimentation in the deciphering of texts throughout history.

In this paper it has been carried out exhaustively for a wide range of languages, around a hundred, and from nine phila, an analysis of whether there is invariance in the frequency of letters and the Index of Coincidence for the various languages to distinguish them from others, even nearby languages.

On the other hand, it had never been considered to analyze whether the test of time ​​substantially modified or not these invariants, since it was not necessary in the historical environments of war and espionage, typical of cryptography and cryptanalysis; a diachronic study that we take into account here.

In this study we make clear that languages ​​are very robust over time and that the Frequency and the I.C. of the letters remain stable despite the passage of centuries.

Another novelty, never previously considered, that our study analyzes, is to apply the same analysis that has been done to letters or graphemes to phonemes, reaching similar conclusions.

Sometimes we have military or civil documents, texts of different themes, colophones, … encrypted, of different length, made several centuries ago, where it is not easy to suppose the source language of the cipher, being necessary to apply the conclusions here obtained. A case where these conclusions have been applied has been the study of a text inscribed in a wood carving around the 14th and 15th centuries, with a total of almost two hundred letters, resulting in a text of proto-Berber origin [130].

The results show that there has been a high diachronic continuity and stabiliy of the language over the centuries. The Index of Coincidence, by its own mathematical definition, is not a very fine discriminator tool, although it is highly stable and very centered in its average value. Refereing to the Frequency, the combination and dual analysis for graphemes and phonemes helps to bring the characteristics of the language together, strengthening its own and distinctive aspects and discriminating one language versus others. Languages have a very strong invariant for their differentiation in the distance of the frequencies of their minimum units (graphemes/phonemes).

## Supporting information

S1 File Raw material of all the texts and languages. (ZIP)

S2 File Maple script for average length of the texts. (MW)

S3 File Maple script for analysing graphemes: Latin and Spanish. (MWS)

S4 File Maple script for analysing phonemes: Latin and Spanish. (MWS)

S5 File Excel script for Figs 2, 5, 8 and 11. (XLSX)

S6 File Maple script for analysing the rest of the languages. (MW)
