# Correction: Graphemic-phonetic diachronic linguistic invariance of the frequency and of the Index of Coincidence as cryptanalytic tools

**DOI:** 10.1371/journal.pone.0215879

**Published:** 2019-04-18

**Authors:** Vicente Jara Vera, Carmen Sánchez Ávila

The Funding Statement is incorrect. The correct Funding statement is as follows: This study has received support from the Instituto Nacional de Ciberseguridad (INCIBE), from Ministerio de Economía y Empresa of Spain, within the framework of the "Ayudas para la excelencia de los equipos de investigación avanzada en ciberseguridad" (ref INCIBEI-2015-27342).
